# Deregulated immune cell recruitment orchestrated by FOXM1 impairs human diabetic wound healing

**DOI:** 10.1038/s41467-020-18276-0

**Published:** 2020-09-16

**Authors:** Andrew P. Sawaya, Rivka C. Stone, Stephen R. Brooks, Irena Pastar, Ivan Jozic, Kowser Hasneen, Katelyn O’Neill, Spencer Mehdizadeh, Cheyanne R. Head, Natasa Strbo, Maria I. Morasso, Marjana Tomic-Canic

**Affiliations:** 1 Laboratory of Skin Biology, National Institute of Arthritis and Musculoskeletal and Skin Diseases, Bethesda, MD 20892 USA; 2 Wound Healing and Regenerative Medicine Research Program, Dr Phillip Frost Department of Dermatology and Cutaneous Surgery, University of Miami Miller School of Medicine, Miami, FL 33136 USA; 3 Biodata Mining and Discovery Section, National Institute of Arthritis and Musculoskeletal and Skin Diseases, Bethesda, MD 20892 USA; 4 Department of Microbiology and Immunology, University of Miami Miller School of Medicine, Miami, FL 33136 USA; 5 John P. Hussman Institute for Human Genomics, University of Miami Miller School of Medicine, Miami, FL 33136 USA

**Keywords:** Inflammation, Skin diseases, Molecular medicine

## Abstract

Diabetic foot ulcers (DFUs) are a life-threatening disease that often result in lower limb amputations and a shortened lifespan. However, molecular mechanisms contributing to the pathogenesis of DFUs remain poorly understood. We use next-generation sequencing to generate a human dataset of pathogenic DFUs to compare to transcriptional profiles of human skin and oral acute wounds, oral as a model of “ideal” adult tissue repair due to accelerated closure without scarring. Here we identify major transcriptional networks deregulated in DFUs that result in decreased neutrophils and macrophages recruitment and overall poorly controlled inflammatory response. Transcription factors *FOXM1* and *STAT3*, which function to activate and promote survival of immune cells, are inhibited in DFUs. Moreover, inhibition of FOXM1 in diabetic mouse models (STZ-induced and db/db) results in delayed wound healing and decreased neutrophil and macrophage recruitment in diabetic wounds in vivo. Our data underscore the role of a perturbed, ineffective inflammatory response as a major contributor to the pathogenesis of DFUs, which is facilitated by FOXM1-mediated deregulation of recruitment of neutrophils and macrophages, revealing a potential therapeutic strategy.

## Abstract (web summary)

Diabetic foot ulcers (DFU) represent a complex disease with limited treatment options. Here, the authors compare human RNASeq patient data from DFU, oral mucosa and skin acute wounds, identifying FOXM1 as a mediator of macrophage and neutrophil recruitment, which contributes to disease pathogenesis and is dysregulated in patients.

## Introduction

Diabetic foot ulcers (DFUs), a well-known and devastating complication of Diabetes Mellitus, represent one of the most prevalent types of chronic wounds. They are a frequent cause of lower limb amputations with a high mortality rate, overall resulting in ~$9–13 billion in health care costs^1,2^. The development of DFUs is multifactorial and is associated with intrinsic factors that include neuropathy, ischemia, infection, impaired immune function, fibrosis, and vascular problems, all of which contribute to poor healing outcome^1,3–5^. DFUs are characterized by a hyperproliferative, non-migratory epidermis with deregulated inflammatory response that contributes to tissue damage, inhibition of epithelialization and unresolved infection^1,6,7^.

The inflammatory response comprises the early response upon injury and plays an essential role that facilitates progression of healing. Recruitment of immune cells to the site of injury coordinates multi-cellular healing response and prevents infection^1,8–10^. Neutrophils are the first inflammatory cells to migrate to the wound to clear dead cells and infectious microorganisms^11–13^. Neutrophil activity must be tightly controlled to prevent excessive inflammation and host tissue damage. It is regulated by apoptosis after clearance of infection^1,12^ as well as induction of local production of cortisol^14,15^. Neutrophils isolated from patients with DFUs were shown to have deregulated process of neutrophil extracellular traps (NETs) activation and release (NETosis) underscoring their role in poor healing^16,17^. Next, monocytes appear at the site of injury and are converted to macrophages^18–21^. Specifically, recruited monocytes are activated in response to microbial products and inflammatory mediators to transition to M1 macrophages, a pro-inflammatory phenotype that aids in preventing infection^18^. As the inflammatory response progresses, macrophages transition to an anti-inflammatory and “pro-healing” M2 phenotype that aids in repair of tissue by releasing various factors, such as AREG, TGF-α, PDGF, and IGF-1^8,18^. Macrophages remove apoptotic neutrophils by phagocytosis and play an important role in augmenting the inflammatory response. They also initiate granulation tissue formation by releasing pro-inflammatory cytokines (IL-1β and IL-6) and growth factors (FGF, EGF, and PDGF)^1,8,12,18^. These processes must be tightly regulated for proper healing to occur^22^.

Acute wound healing proceeds through multiple overlapping phases that include hemostasis, inflammatory, proliferative, and remodeling phases^1^. Inflammatory response is considered an “engine” that activates the repair process and, due to its potential damaging effects, is very tightly regulated^1^. Oral wounds represent a paradigm of an “ideal” prototype of adult tissue repair due to its intrinsic ability for accelerated wound healing without scar formation^23–25^. Studies have investigated the mechanisms of rapid oral wound healing in various in vitro and animal studies focusing on inflammation, proliferation, and migration capacity of keratinocytes^23,26^. We recently characterized a unique transcriptional network regulated by the *SOX2* and *PITX1* transcription factors that primes human oral wounds for rapid wound healing^23^. The priming of oral tissue for accelerated healing is linked to higher baseline inflammatory mediators gearing for rapid tissue repair. Furthermore, we found that *SOX2* accelerated wound healing by promoting keratinocyte migration and angiogenesis through upregulation of EGFR ligands and subsequent activation of ERK/MAPK pathway^26^. In contrast to acute cutaneous and oral wounds, DFU healing does not progress through phases and is characterized by a stalled undefined non-healing state that includes deregulated inflammation that is considered less effective to facilitate progression of healing, decreased angiogenesis, hyperproliferative non-migratory epithelium, lack of response to growth factors, and fibrosis.

Distinct inflammatory responses in oral and acute cutaneous wounds have been previously described^23^. In contrast to skin, oral mucosa has a heightened inflammatory response present at baseline^23^. Even though the oral mucosa is primed with a heightened inflammatory response, resolution of inflammation rapidly occurs to prevent chronic inflammation. Although the inflammatory response has major benefits during wound healing, adverse consequences associated with a chronic inflammatory response have been recognized. Thus, chronic, unresolved inflammation is a hallmark of chronic, non-healing wounds and has detrimental effects on the wound-healing process^1,6,7,27^. We have shown that prolonged inflammation in chronic wounds is present at suboptimal levels and insufficient to facilitate progression of healing compared to normal acute wound inflammatory response^6,7^. Furthermore, it has been shown that transition of M1 to M2 macrophages appears to be impaired in diabetic wounds^28,29^. Therefore, maintaining an appropriate balance, level and timing of the inflammatory response is essential to ensure proper healing outcome.

This study reports a transcriptomic RNA-seq data of wound edge biopsies derived from 13 DFU patients and diabetic foot skin (DFS). Comparative profiling to human acute oral and skin healing wound transcriptomes identified specific molecular mechanisms and transcriptional networks that are deregulated in DFUs. Our results show that a previously undescribed inflammatory transcriptional signature present in acute oral and skin wounds involved in promoting cell proliferation and immune-cell recruitment is deficient in DFUs. In addition, we determine an immune-cell profile in which activation and recruitment of macrophages and neutrophils is absent in DFUs. Furthermore, FOXM1, responsible for activation and recruitment of inflammatory cells, was found downregulated in patients. Using patients as an in vivo approach has some limitation^30^, due in part to the inability to knock-in and -out target genes in patients. Thus, we utilized a combinatorial approach in which we mimic the condition found in DFU patients (FOXM1 downregulation) in diabetic mouse models. These functional analyses of FOXM1 inhibition in diabetic mouse wound models support our findings in DFUs, and show delayed wound healing and suppression of neutrophil and macrophage recruitment to the wound site. The crucial importance of the study is that it provides insights into mechanistic aspects of a disease that has had many futile therapeutic trials in the past two decades. These results demonstrate that a deregulated immune response in which impaired activation, recruitment and survival of immune cells mediated by downregulation of FOXM1 contribute to the pathogenesis of DFUs. Taken together, identification of FOXM1-mediated deficient immune-cell recruitment identifies targets for development of therapies aimed at reprogramming of chronic, non-healing DFUs into healing-competent wounds.

## Results

### DFUs and oral mucosa share similar wound-activated transcriptional signatures

**Fig. 1:**
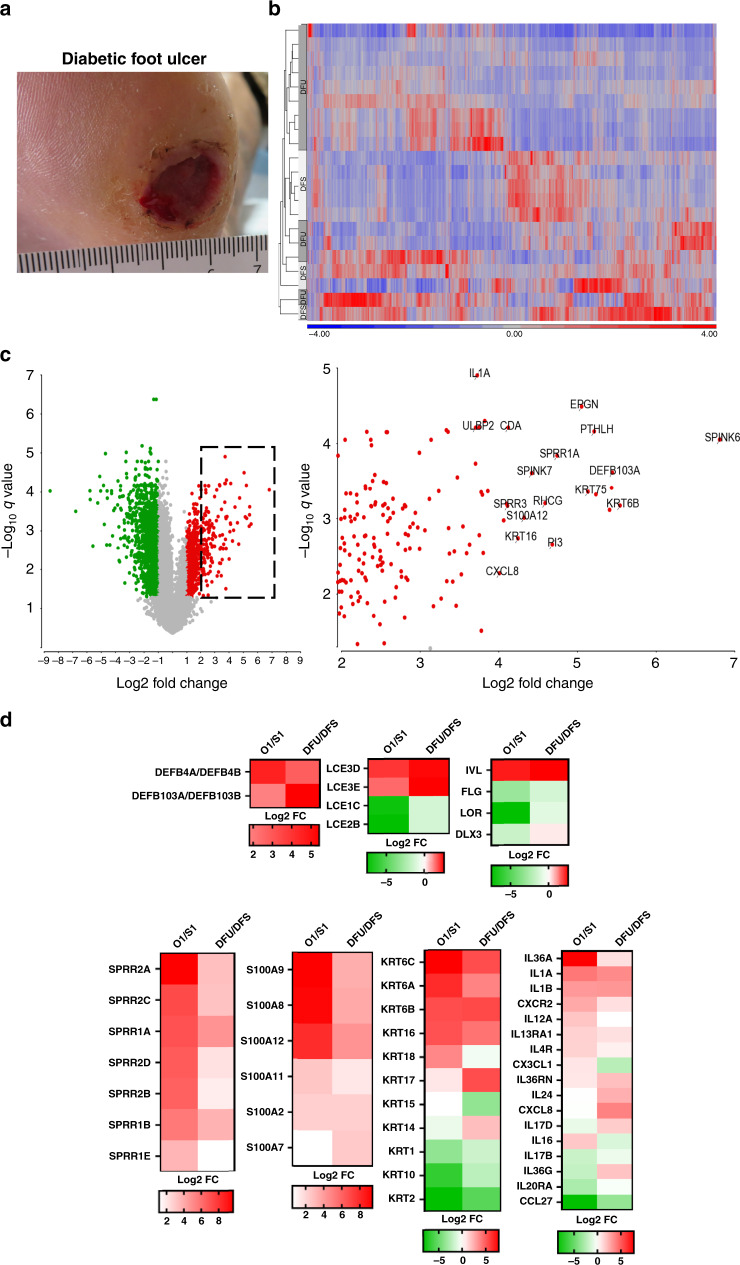
Wound-activated gene signature found at baseline of oral mucosa is activated in DFUs. **a** Representative image of a diabetic foot ulcer (DFU). **b** Heatmap of genes regulated in DFUs. **c** Volcano plot indicating differentially regulated genes in DFUs. Dotted line region is magnified on the right panel highlighting some of the most significantly upregulated genes in DFUs. **d** Wound-activated gene signature of a subset of genes involved in epidermal differentiation, intermediate filaments and inflammatory cytokines shows similarities between baseline oral mucosa and DFUs.

We have shown that the oral mucosa has a unique gene signature that resembles wound-activated transcriptional networks^23^. Since the oral mucosa has an intrinsic ability for rapid wound healing, we postulated that this wound-activated gene signature is deregulated in DFUs. To this end, we performed RNA-seq analysis on tissue biopsies from patients with DFUs (*n* = 13; GSE134431) (Fig. 1a, b). Transcripts up- and downregulated in DFUs were found to be associated with the wound-activated signature (Fig. 1c and Supplementary Fig. 1). We compared DFUs to unwounded oral day 1 wound-activated signatures and found similar signatures of gene families involved in differentiation, intermediate filament components and inflammatory cytokines (Fig. 1d). The differentiation markers (*LCEs*, *IVL*, *FLG*, *LOR*, and *DLX3*) showed similar levels of activation in oral and DFUs. In addition, we found *SPRR* and *S100* cluster of genes induced in oral mucosa and DFUs, although oral mucosa showed stronger induction when compared to DFUs. Wound-activated keratin genes (*Krt6, Krt16)* also showed a significant level of activation. In contrast, *Krt17* was found to be significantly enriched in DFUs when compared to oral mucosa. Differentiation-associated keratins (*Krt1, Krt10*, and *Krt2*) were downregulated in both oral mucosa and DFUs. Inflammatory cytokines were found to be similarly regulated (Fig. 1d). These findings indicate that oral mucosa (presumably a tissue “primed” for rapid healing) and DFUs share some common pathways of wound-activated signature that provide a rational basis for potential reprogramming of chronic into a healing tissue. Furthermore, these findings suggest that there are alternate transcriptional networks deregulated in DFUs that contribute to the non-healing phenotype.

### Deregulation of the inflammatory response in DFUs

**Fig. 2:**
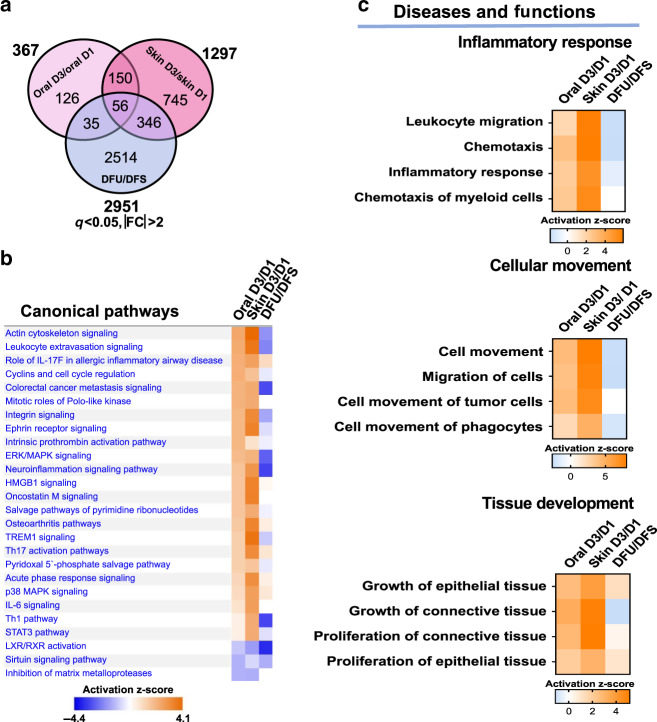
Pathways and functions involved in inflammatory response and cellular movement are inhibited in DFUs but activated in oral and skin wounds. **a** Venn diagram of significantly regulated genes in oral day 3 (D3)/day 1 (D1), skin D3/D1, and DFU/DFS (diabetic foot skin). **b** Top canonical pathways and **c** diseases and functions found to be enriched in oral D3/D1, skin D3/D1, and DFU/DFS showing downregulation of cellular movement and inflammatory response in DFUs.

To investigate the transcriptomic differences between wounding in healthy-healing capable tissues (human oral mucosa and skin acute wounds) and chronic, non-healing DFUs, we examined the significant differentially expressed genes in paired oral and skin acute wounds at day 3 wounds in comparison to DFUs. We identified 367 and 1297 genes differentially regulated in oral and skin wounds, respectively, compared to 2951 genes differentially regulated in DFUs (Fig. 2a). There were only 56 genes common between all three groups. Of note, there are 346 genes common between acute human skin wound and DFUs. Ingenuity Pathway Analysis (IPA) of the differentially expressed genes identified multiple enriched pathways, with a significant number involved in the inflammatory response and cell movement processes. Some of the top enriched pathways corresponded to actin cytoskeletal signaling, leukocyte extravasation signaling and cell-cycle regulation (Fig. 2b), all of which play important roles in the wound-healing process^1^. Strong activation of these pathways in oral and skin day 3 acute wounds was inhibited in DFUs (Fig. 2b). In contrast to suppression found in DFUs, gene ontology (GO) analysis highlighted a strong enrichment in processes involved in the inflammatory response, cell movement and tissue development, with oral and skin day 3 wounds showing robust activation of these processes (Fig. 2c and Supplementary Fig. 2). The top processes enriched in the inflammatory response corresponded to leukocyte migration, chemotaxis and chemotaxis of myeloid cells. We conclude that the inflammatory signature present in oral and skin acute wounds that facilitates healthy-wound healing is downregulated in chronic non-healing DFUs.

**Fig. 3:**
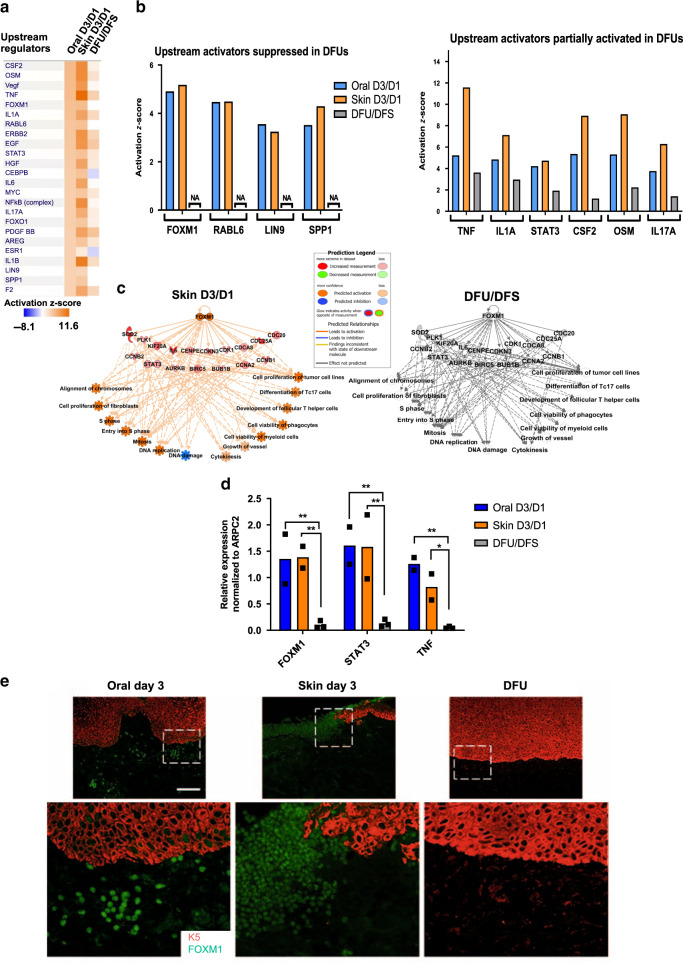
Upstream regulators involved in promoting proliferation and cell survival of immune cells are inhibited in DFUs and activated in oral and skin acute wounds. **a** Top upstream regulators enriched in oral D3/D1 vs. skin D3/D1 vs. DFU/DFS. **b** Upstream regulators found to be activated in oral and skin wounds that are suppressed or partially regulated in DFUs involved in proliferation, inflammatory response, leukocyte migration and proliferation of leukocytes. NA non applicable. **c** FOXM1 predicted network shows activation of proliferation and inflammatory response in oral and skin wounds compared to suppression in DFUs. **d** qPCR validations of upstream regulators FOXM1, STAT3, and TNFα confirms suppression in DFUs compared to activation in oral and skin human wounds. *n* = 2 biologically independent samples for oral and skin wounds; *n* = 3 biologically independent samples for DFUs. Data presented as mean ± SD. FOXM1 *P*-values oral D3/D1 vs DFU/DFS: **P = 0.0095; FOXM1 *P*-values skin D3/D1 vs DFU/DFS: **P = 0.008; STAT3 *P*-values oral D3/D1 vs DFU/DFS: **P = 0.003; STAT3 *P*-values skin D3/D1 vs DFU/DFS: **P = 0.0034; TNF *P*-values oral D3/D1 vs DFU/DFS: **P = 0.0041; TNF *P*-values skin D3/D1 vs DFU/DFS: *P = 0.0436 (two-way ANOVA followed by Tukey’s post hoc test). **e** Immunostaining of FOXM1 (green immunofluorescence signal) and keratin 5 (K5, in red) in oral and skin day 3 wounds and DFUs. Robust staining of FOXM1 found in oral and acute skin wounds but is absent in DFUs. Stainings were performed once with three biologically independent patient samples per group. Scale bar = 100 µm.

Next, we performed IPA analysis to determine specific upstream regulators responsible for such inflammatory response signatures (present in oral and skin acute wounds but deregulated in DFUs) (Fig. 3a). We grouped upstream regulators based on those activated in oral and skin acute wounds and those that are either suppressed or partially activated in DFUs (Fig. 3b). Several genes involved in cell proliferation and cell survival (*FOXM1*, *RABL6*, *LIN9*, and *SPP1*) were suppressed in DFUs in contrast to being found strongly activated in oral and skin wounds. In addition, we identified a set of growth factors, cytokines and transcription factors associated with the inflammatory response (*TNFα*, *STAT3, IL1A, CSF2*, *OSM*, and *IL17A*) strongly activated in oral and skin acute wounds to be significantly less activated in DFUs. Moreover, networks connecting upstream regulators to downstream biological processes predicted to be significantly activated in oral and skin acute wounds, were found suppressed in DFUs (Fig. 3c and Supplementary Fig. 3). Among them was *FOXM1*, a transcription factor associated with promoting proliferation and cell viability of inflammatory cells (Fig. 3c). To further validate our findings and confirm expression of upstream regulators in oral, skin, and DFUs we used qPCR and immunostaining (Fig. 3d, e and Supplementary Fig. 4). As expected, we found *FOXM1, STAT3*, and *TNFα* to be induced in oral and skin acute wounds. However, they were found significantly suppressed in DFUs, confirming our transcriptomic analyses.

We further reinforced the global role of FOXM1 in physiologic wound healing by examining expression of its targets and regulators in acute oral and skin wounds. 50/54 of FOXM1’s transcriptional target genes were commonly regulated in both oral and skin wounds at post-wounding day 3 (Supplementary Fig. 5a). Moreover, we found that ZBTB17, a negative regulator of FOXM1, was suppressed in acute wounds (Supplementary Fig. 5b), further supporting FOXM1’s activation in this context. On the other hand, several FOXM1-interacting proteins involved in stimulating wound healing (e.g., IL-6, MMP9, and SOD2)^31–34^ were downregulated in DFUs compared to oral and skin acute wounds (Supplementary Table 1), demonstrating pathologic suppression of FOXM1 pathway in DFUs. Next, we evaluated the time course of FOXM1 expression and its antagonist, FOXO1 (Supplementary Fig. 6). We found FOXM1 expression levels peak at day 3 post wounding in oral wounds, whereas its expression peaks at day 6 in skin wounds. In contrast, DFUs show decreased FOXM1 expression. FOXO1 expression was constitutively present, but not regulated in oral and skin wounds. It was slightly induced in DFUs, suggesting that FOXO1 may contribute to FOXM1 inhibition. Taken together these results emphasize downregulation of the inflammatory response in DFUs when compared to the transcriptional signature of acute oral and skin wounds.

### Altered immune-cell activation in DFUs

**Fig. 4:**
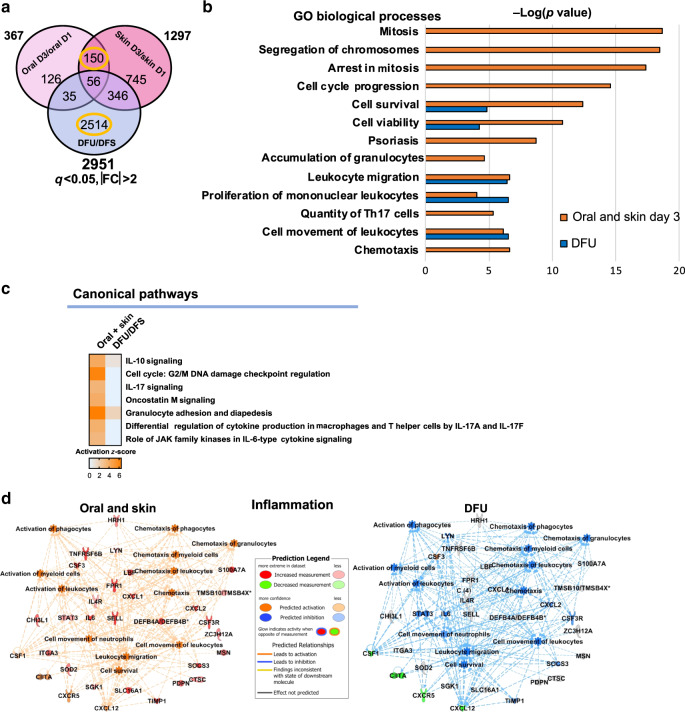
Inflammatory signature is inhibited in DFUs. **a** Venn diagram of significantly regulated genes from oral, skin, and DFUs. **b** Top enriched GO processes from commonly regulated genes in oral and skin wounds compared to DFU specific genes (circled in yellow in part **a**) demonstrates processes involved in cellular proliferation and inflammation to be deregulated in DFUs compared to acute wounds (oral and skin). **c** Top canonical pathways involved in inflammation and **d** IPA-predicted network of inflammation in oral and skin compared to DFUs (*n* = 8 biologically independent samples) shows inhibition of cell proliferation and inflammation in DFUs compared to acute wounds (oral and skin).

Next, we investigated the transcriptional networks commonly regulated in healthy, acutely-healing tissues that are absent in DFUs. To this end, we compared the shared 150 genes regulated in oral and skin day 3 wounds to the 2514 genes that are unique to DFUs (Fig. 4a).

Enriched GO biological processes in oral and skin day 3 wounds included mitosis, cell-cycle progression, cell viability, accumulation of granulocytes, proliferation of mononuclear leukocytes, and chemotaxis, were either found absent or mildly regulated in DFUs (Fig. 4b) with the exception of leukocyte migration. Furthermore, IPA-predicted activation of key pathways involved in regulation of cell proliferation and the inflammatory response (Fig. 4c) and networks connecting genes to downstream biological processes involved in inflammation and cell proliferation (Fig. 4d and Supplementary Fig. 7a) in acute oral and skin wounds and their suppression in DFUs. The significant activation of inflammation in oral and skin acute wounds was strongly downregulated in DFUs (Fig. 4d), indicating that the unique oral and skin inflammatory signature responsible for stimulating progression of healing is suppressed in DFUs. In addition, cell proliferation was predicted to be markedly suppressed in DFUs (Supplementary Fig. 7a). Given that hyper-proliferation of the epidermis is a hallmark of DFUs, we reasoned that the suppressed proliferation in DFUs predicted by IPA was specific to immune cells, rather than epidermal cells. To assess proliferation of immune cells in oral, skin, and DFU wounds, we performed immunohistochemistry staining for PCNA and CD68, a marker of macrophages. We found decreased PCNA and CD68 co-staining in DFUs compared to oral and skin wounds, demonstrating decreased immune-cell proliferation (Supplementary Fig. 7b). This was further corroborated with Ki67 staining (Supplementary Fig. 8). These results support the notion of suppressed proliferation and viability of immune cells that contribute to the inefficient inflammatory response in DFUs.

**Fig. 5:**
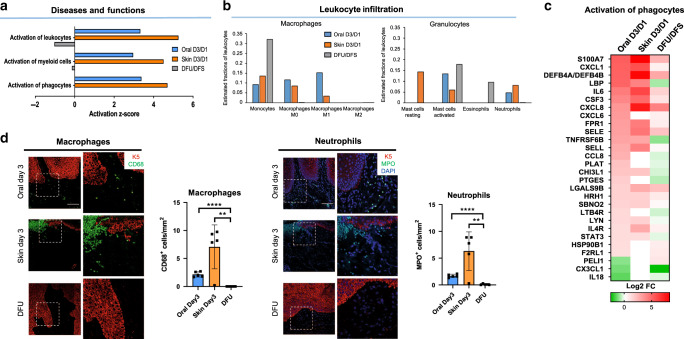
Deregulated activation of immune cells in DFUs. **a** Top functions enriched in the inflammatory response demonstrating inhibition of immune-cell activation in DFUs. **b** Prediction of estimated proportions of a subset of leukocytes (macrophages and granulocytes) in oral, skin, and DFUs based on gene expression demonstrates decreased macrophage activation and neutrophil recruitment with increased eosinophils in DFUs. **c** Subset of genes involved in activation of phagocytes showing downregulation of several genes in DFUs (*n* = 8 biologically independent samples). **d** Representative pictures of oral and skin day 3 wounds and DFUs show basal keratin marker K5, macrophage marker, and neutrophil marker MPO. Quantification of *n* = 5 biologically independent samples demonstrates decreased macrophage activation (**P = 0.0038, ****P ≤ 0.0001) and neutrophils (**P = 0.0048, ****P ≤ 0.0001) in DFUs compared to oral and skin wounds. Data presented as mean ± SD (two-tailed unpaired Student’s *t*-test). Scale bar = 100 µm.

Next, we focused on understanding the deregulated inflammatory response in DFUs. Activation of leukocytes, activation of myeloid cells and activation of phagocytes was found enriched and strongly activated in oral and skin acute wounds but suppressed in DFUs (Fig. 5a). Investigation of the immune-cell landscape using CIBERSORT algorithm^35,36^ showed that DFUs had a high proportion of monocytes, indicating their active recruitment. We found macrophages to be absent in DFUs, further supporting deregulated activation of immune cells in DFUs. Oral and skin acute wounds showed M1 macrophages to be present at day 3 post wounding, whereas M2 macrophages were present at day 6 (Fig. 5b and Supplementary Fig. 9). Moreover, we identified neutrophils to be present in oral and skin acute wounds, but absent in DFUs. Eosinophils were predicted to be present only in DFUs, whereas mast cells were detected in oral, skin acute wounds, and in DFUs (Fig. 5b). Mast cells showed no differences in recruitment in oral, skin or DFUs, whereas eosinophils were only present in DFUs and absent in oral and skin (Fig. 5b).

T cells, B cells, dendritic cells, and NK cells were also found to be present (Supplementary Fig. 10). To further confirm the predicted immune-cell landscape, we generated a heatmap of differentially regulated genes involved in activation of phagocytes. Several genes involved in activation of phagocytes were found to be suppressed in DFUs compared to oral and skin wounds (Fig. 5c), further supporting deregulation of activation of immune cells in DFUs and inability to clear infection. We further validated these findings by immunohistochemistry and confirmed presence of neutrophils in oral and skin day 3 wounds as well as their absence in DFUs (Fig. 5d). CD68 and p-STAT3 immunostaining showed presence and activation of macrophages in oral and skin wounds, but not in DFUs (Fig. 5d). These results confirm decreased immune-cell activation in the DFU environment.

### Inhibition of FOXM1 delays wound healing and suppresses the inflammatory response in vivo

**Fig. 6:**
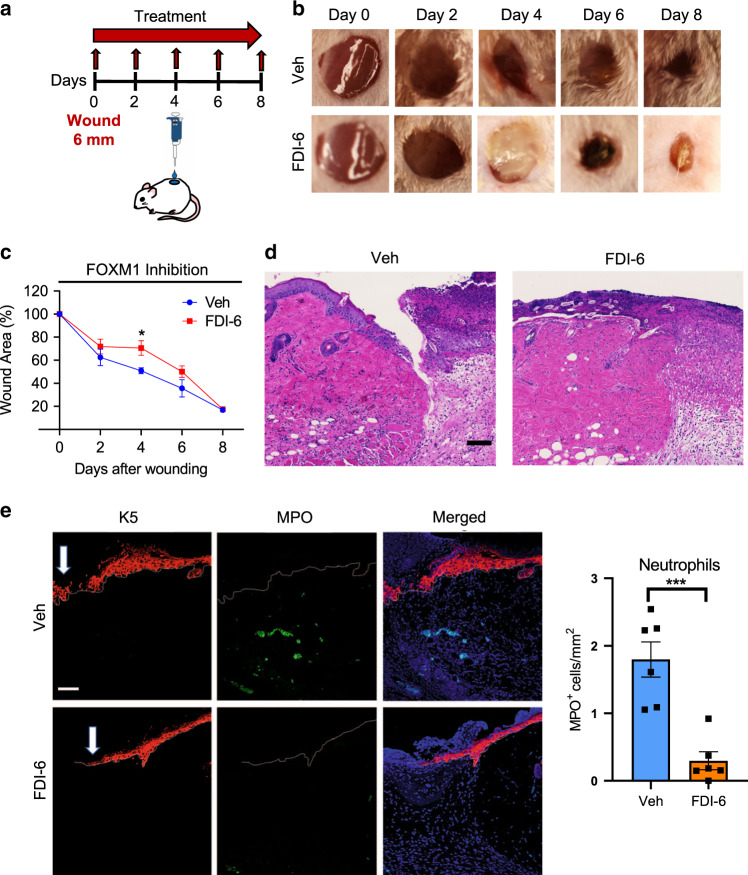
Inhibition of FOXM1 suppresses immune-cell response and inhibits wound healing in vivo. **a** Schematic of in vivo wound-healing assay. CD1 (non-diabetic) mice were wounded and treated topically with either the FOXM1 inhibitor FDI-6 or vehicle every other day for 8 days. **b** Representative images of wounded skin after topical treatment with either vehicle or FDI-6 at 0, 2, 4, 6, and 8 days after wounding. **c** Percent of wound area at each time following vehicle or FDI-6 treatment relative to the original wound area. Quantification of wound areas in *n* = 6 (Veh) and 8 (FDI-6) wounds per group were performed with Fiji software. Data presented as mean ± SEM. *P = 0.042 (two-tailed unpaired Student’s *t* test). **d** H&E staining of day 4 wounds demonstrating decreased immune cell infiltrates in FDI-6 treated wounds compared to vehicle control wounds. **e** Representative pictures of vehicle and FDI-6 treated wounds at day 4 show basal keratin marker K5, and neutrophil marker MPO. Treatment of wounds with FDI-6 resulted in decreased neutrophils compared to vehicle treated wounds. *n* = 3 animals per group examined over two independent experiments. Data presented as mean ± SD. ***P = 0.0005 (two-tailed unpaired Student’s *t* test). White arrows indicate the wound edge of the migrating epithelial tongue. Scale bar = 100 µm.

To corroborate data from patients, we investigated the effect of FOXM1 inhibition on wound healing in vivo. We utilized a pharmacological approach using a FOXM1 specific inhibitor, FDI-6, that was topically applied to full-thickness wounds created on dorsal skin of mice (Fig. 6a). FDI-6 acts by blocking FOXM1 ability to bind DNA, suppressing transcription of FOXM1 target genes^37^. We compared the kinetics of wound healing in wounds treated with FDI-6 and vehicle treatment served as a control. Treatment with FDI-6 significantly inhibited wound healing compared to vehicle at day 4 post-wounding (Fig. 6b, c), the time point of the peak inflammatory response. We found that neutrophils and macrophages were the only immune cell types present in acute wounds but absent in DFUs (Fig. 5b). Thus, we postulated that FOXM1 may be regulating these immune-cell populations. To test effects of FOXM1 inhibition on neutrophils in vivo, we assessed the presence of neutrophils in day 4 wounds treated with either vehicle or FDI-6. Hematoxylin and eosin staining of day 4 wounds treated with FDI-6 revealed decreased immune cell infiltrates compared to vehicle (Fig. 6d). MPO staining showed decreased presence of neutrophils in wounds treated with FDI-6 compared to vehicle (Fig. 6e).

**Fig. 7:**
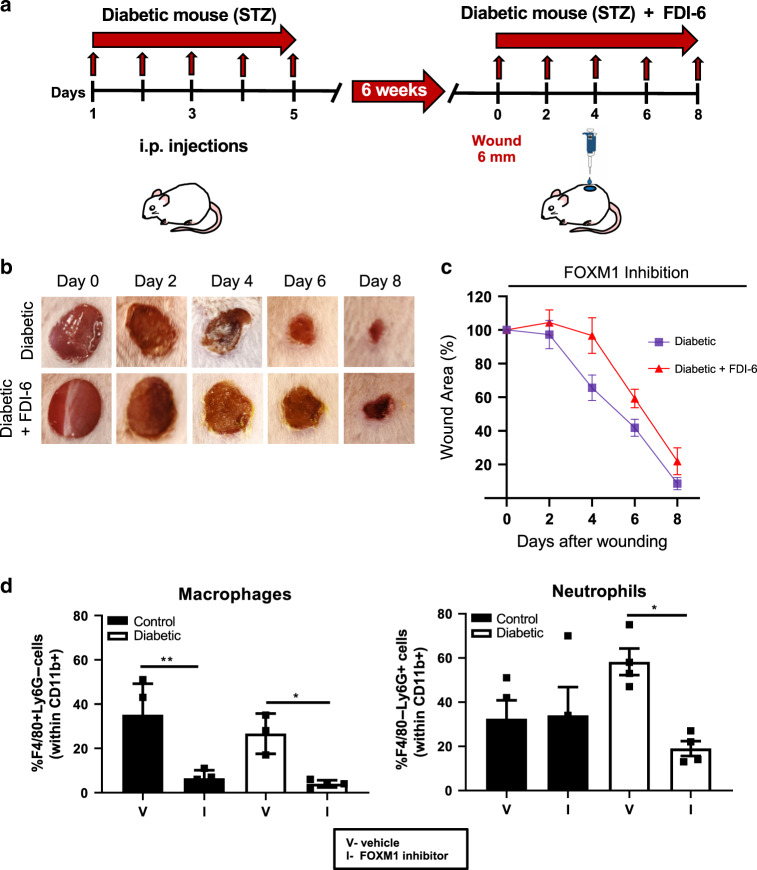
Inhibition of FOXM1 further impairs wound healing and decreases frequency of macrophages and neutrophils in the wounds of diabetic mice. **a**. Schematic of in vivo wound-healing assay in STZ-induced diabetic mice. CD1 mice were i.p. injected with STZ to induce diabetes and were maintained for 6 weeks for effects of diabetes on wound healing to occur. Mice were wounded and treated topically with either the FOXM1 inhibitor FDI-6 or vehicle every other day for 8 days. **b** Representative images of wounded skin after topical treatment with either vehicle or FDI-6 at 0, 2, 4, 6, and 8 days after wounding. **c** Percent of wound area at each time following vehicle or FDI-6 treatment relative to the original wound area. Quantification of wound areas in *n* = 10 (diabetic) and *n* = 16 (diabetic+FDI-6) wounds per group were performed with Fiji software. Data presented as mean ± SEM. **P = 0.0053 (two-way ANOVA followed by Tukey’s post hoc test). **d** Wound edge skin at day 4 was collected and frequencies of macrophages (F4/80 + Ly6G−) and neutrophils (F4/80-Ly6G+) within gated myeloid cells, CD11b+ cells were quantified by flow cytometry. Data represent 4 wounds per group. Data presented as mean ± SEM. **P = 0.0028 for control group, *P = 0.023 for diabetic group for macrophages; *P = 0.027 for diabetic group for neutrophils, as calculated using one-way ANOVA with Tukey’s multiple comparisons test.

We next investigated the effects of FOXM1 using the streptozoticin (STZ)-induced diabetic mouse model. Full-thickness wounds were created on the dorsal side of mice and topically treated with FDI-6 or vehicle 6 weeks after STZ intraperitoneal injections (Fig. 7a). We compared the kinetics of wound healing in diabetic mice wounds treated with FDI-6 and vehicle treatment (control). As expected, diabetic wounds showed a delay in healing compared to non-diabetic control wounds. Treatment of FDI-6 further delayed wound healing in diabetic mice compared to vehicle treated diabetic wounds (Fig. 7b, c). To test effects of FOXM1 regulation of neutrophil and macrophage infiltration in diabetic wounds, we performed FACS analysis in two types of diabetic wounds, STZ and db/db. We found overall significant decrease in myeloid CD11b^+^ cells (Supplementary Figs. 12 and 14). Treatment with FOXM1 inhibitor resulted in significant decrease of neutrophils and macrophages in both types of diabetic wounds treated with FDI-6 (Fig. 7d and Supplementary Figs. 11 and 13). Although non-diabetic control mice treated with FDI-6 did not show any significant changes in neutrophils by FACS, we observed significant decrease of neutrophils in diabetic wounds treated with FDI-6 in both diabetic mouse models, STZ and db/db. These findings confirm FOXM1 as a regulator of the inflammatory response during wound healing that is suppressed in DFUs, thus contributing to deregulated inflammatory response and overall non-healing chronic wound phenotype in diabetic patients.

## Discussion

**Fig. 8:**
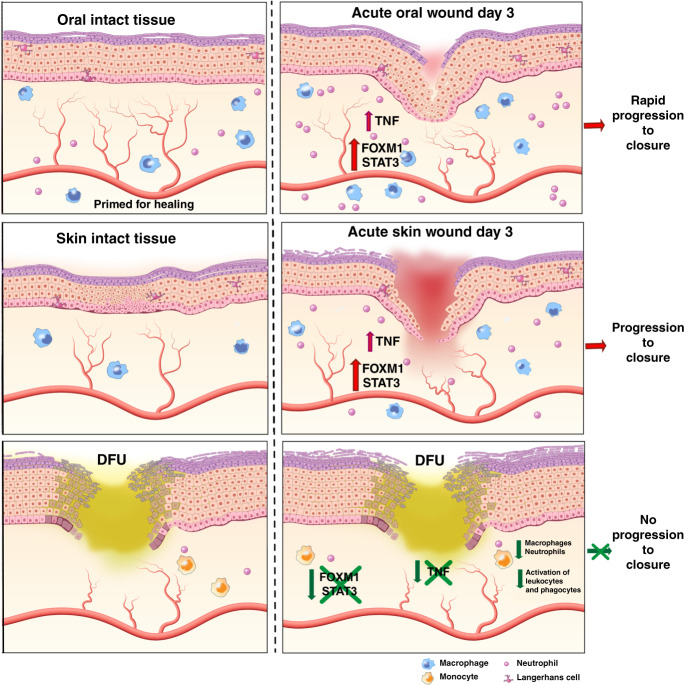
The diagram summarizes our findings and shows a model of unwounded oral mucosa, skin, and DFUs. It demonstrates similar wound-activated signature of genes involved in differentiation, cytokines, and intermediate filaments. Inhibition of FOXM1, STAT3, and TNFα regulators results in lack of immune-cell activation, proliferation and survival in the DFU environment contributing to deregulated inflammatory response and inhibition of wound healing.

In this study, we performed a comprehensive comparative analysis between tissue biopsies derived from patients with DFUs and paired human oral mucosa and skin acute wounds using next-generation sequencing and IPA. We identified previously undescribed transcriptional networks responsible for activation, proliferation, and survival of immune cells to be suppressed in DFUs. In addition, we determined a specific inflammatory and immune-cell signature characteristic for DFUs. Our data demonstrate that lack of immune-cell activation and survival in the DFU environment leads to decreased presence of macrophages and neutrophils contributing to inhibition of wound healing (Fig. 8).

The oral mucosa is recognized for its ability for rapid healing without scar formation. Our previous findings demonstrated that the oral mucosa has a transcriptional signature that primes this tissue to rapidly respond to injury^23,26^. The priming of oral mucosa for healing is presented by a wound-activated gene signature at baseline, which allows for the more rapid wound-healing response, when compared to cutaneous wounds. Interestingly, DFUs shared similarities with the wound-activated gene signature of oral mucosa. Of note, these signatures are present in the oral mucosa tissue prior to injury and are further activated by wounding^23,26^, whereas they represent a maximal tissue response in non-healing DFUs. Furthermore, there are 356 common genes found between acute human skin wound and DFUs. This is not surprising since one can expect a core signature of a “wound” phenotype to be shared. Moreover, the presence of these signatures indicates DFU’s potential capacity to implement a wound-activated phenotype upon potential therapeutic intervention. Our analysis also showed enriched canonical pathways and functions in oral and skin wounds that are significantly suppressed in DFUs, including actin cytoskeleton signaling, leukocyte extravasation signaling, integrin signaling, and the inflammatory response. Therefore, although DFUs may be able to activate some aspects of a wound-healing phenotype, this is incomplete as they are unable to properly execute a set of essential pathways and cellular functions, delineating what contributes to impairment of healing.

Upstream regulators that were either suppressed or less activated in DFUs identified transcription factors *FOXM1* and *STAT3* as well as the cytokines *TNF, IL1A, CSF2*, *OSM*, and *IL17A*. The *FOXM1* gene is a member of the Forkhead superfamily of transcription factors and has been shown to promote activation, proliferation, and survival of immune cells as well as prevent DNA damage^38–40^. Our previous findings have demonstrated that microbial-induced DNA damage contributes to a deregulated inflammatory response in DFUs^6^. In addition, *FOXM1* has been shown to be induced by cytokines such as *CSF2*^41^, which was found partially activated in DFUs. *CSF2* has been shown to be increased in wounded skin and plays an important role during inflammation by increasing the number of neutrophils and enhancing their function at the wound site^42,43^. In addition, *CSF2* promotes the activation and survival of macrophages and neutrophils^44,45^. Conversely, diminished *FOXM1* and *CSF2* contribute to lack of immune-cell proliferation and survival in the DFU environment.

We found distinct time control in expression of FOXM1 that peaks at day 3 in oral and at day 6 in skin wounds. This suggests that FOXM1 is integral component of the rapid healing response of the oral mucosa and is in agreement with our previous findings of oral wounds exhibiting a heightened inflammatory response compared to skin wounds^23^. Interestingly, FOXO1 that can antagonize FOXM1 was constitutively present, but not regulated in oral or skin wounds whereas it was found slightly induced in DFUs, suggesting that it may contribute to FOXM1 inhibition. Such potential mechanism is currently under investigation. The functional role of inhibition of FOXM1 (found in DFU patients) was confirmed using diabetic mouse wound models in vivo. As expected, we found that inhibition of FOXM1 resulted in significantly delayed healing and decreased presence of neutrophils and macrophages at the peak of inflammatory response, 4 days post-wounding. Based on our findings, inhibition of FOXM1 further delayed healing in the context of diabetes, suggesting that the presence of diabetes may increase the susceptibility to FOXM1 inhibition. Recent findings have demonstrated that suboptimal inflammatory response in chronic wounds does not reach the level of acute wounds resulting in lingering, ineffective inflammation that halts progression of healing^6,7^. Although DFUs are a complex multifactorial disease with no single cause, our data demonstrate suppression of FOXM1 delays healing, contributing to the phenotypic switch from acute to chronic insufficient healing. Furthermore, the FOXM1-interacting proteins involved in stimulating wound healing (e.g., IL-6, MMP9, and SOD2)^31–34^ were downregulated in DFUs demonstrating pathologic suppression of FOXM1 pathway in DFUs. Thus, we propose that downregulation of FOXM1 correlates with reduced immune cell infiltration into the wound, contributing to the pathophysiology of DFUs.

Given that this is a patient-driven study, we were unable to use traditional approaches of genetic or chemical induction or repression of FOXM1 in patients. Although STZ-induced and db/db models are universally recognized as models to study delayed wound healing, the limited translation to human condition is a recognized limitation^46^, which is why we used a combinatorial approach of primarily human study supported by mechanistic insights from diabetic mouse models. Our in vivo findings in non-diabetic and different diabetic mouse models (STZ-induced and db/db) document that use of a pharmacological approach to selectively inhibit FOXM1 triggers a delay in wound healing and inhibits the recruitment of inflammatory cells to the wound. Additional analysis shows that the expression pattern of ZBTB17, an upstream inhibitor of FOXM1, is consistent with our findings in patient data. Altogether, we provide important mechanistic insights that demonstrate FOXM1 as a regulator of the wound-healing process and corroborates data from patients with DFU.

We also found that *STAT3*, a known regulator of wound healing and activator of immune cells^47–50^, was only partially activated in DFUs compared to oral and skin acute wounds. Decreased activation of p-STAT3 confirmed at the protein level further signifies impaired activation of immune cells in DFUs. Therefore, deregulation of transcriptional networks that results in lack of activation, proliferation, and viability of immune cells in the DFU environment, contributes to the chronic, non-healing wound phenotype, which is unable to reach acute level of inflammation required for progression of healing. As a result, a wound is “stalling”. These findings provide a paradigm shift as increased levels of inflammatory cell infiltrates, increase of pro-inflammatory cytokines such as TNFα and persistence of prolonged inflammation were long thought to be major contributors to delayed healing in chronic wounds^51–53^. Our findings demonstrate the presence of low-potency inflammation whereby the immune cells activation and function is decreased providing inadequate inflammatory response that cannot facilitate progression of healing.

Based on our analysis of the immune-cell landscape, neutrophils are almost completely absent in DFUs compared to oral and skin wounds. Neutrophils are involved in eliminating infection by killing invading microbes and activating other immune cells such as macrophages to aid in healing^11,12^. However, excessive neutrophil activity can potentially lead to tissue damage and inhibition of healing through NETosis^11,12,54^. In addition, it has been shown that diabetes primes neutrophils to undergo NETosis in diabetic human and mouse models to inhibit wound healing^17^. In diabetic mouse models, neutrophils released extracellular traps composed of decondensed chromatin with cytotoxic proteins and induced tissue damage, while inhibiting NETosis accelerated wound healing^17^. Our data demonstrate that neutrophils are decreased in patients’ DFUs at the ulcer edge due to deregulation of transcriptional networks that promote survival of immune cells. It is tempting to postulate that lack of these transcriptional networks may be involved in priming neutrophils to undergo NETosis. This will require further investigation in the context of human non-healing DFUs. However, it is also possible that NETosis may be characteristic of neutrophils residing in the wound bed of DFUs, whereas observed lack of neutrophils at the wound edge further contributes to the inability to respond to pathogens.

Macrophages were also found to be deregulated in DFUs. We found high proportions of monocytes in DFUs, indicating that monocytes are actively recruited to the site of the ulcer. However, we did not detect activated macrophages, indicating impaired activation in DFUs. Macrophages play a crucial role in properly executing the inflammatory phase of wound healing^20,55^ Studies on macrophages during skin repair demonstrated macrophages exert distinct functions during wound healing that are important for controlling the natural sequence of skin repair^20,55^. Emerging evidence have suggested that macrophage dysfunction is associated with poor healing^22,28,29,56^. Macrophage efferocytosis dysfunction has been demonstrated in diabetic mice leading to prolonged inflammatory response and delayed wound healing^28^. Macrophages undergo a phenotypic shift from M1 to M2 type which assures resolution of inflammation and progression of wound healing, while this process is found deregulated in diabetic ulcers resulting in lack of M2 type and accumulation of dysfunctional M1 macrophages^29,57^. In contrast, we found lack of total macrophage population in non-healing DFUs, similar to diabetic mouse wounds treated with FOXM1 inhibitor. The absence of macrophages is not likely to be due to sampling as the tissue obtained from the DFUs in this study was from the wound edge location, with confirmed depth comprising dermis and appropriate quality assessment. Furthermore, presence of keratinocytes at the wound edge and their pro-inflammatory signals in acute wounds provide important “call to arms” resulting in significant immune cell infiltrate at the edge of the wound. Thus, depletion of macrophages and suppression of transcriptional networks that promote activation and survival of immune cells provide important insights into the cellular milieu of the DFU environment and specific non-healing wound phenotype. Based on our in vivo mouse data combined with findings in DFUs, decrease in activated macrophages can be attributed to downregulation of FOXM1.

Our data demonstrate decreased macrophages and neutrophils in DFUs; deregulated recruitment and/or improper functioning of these immune cells may contribute to delayed healing of these wounds. During physiologic wound healing, neutrophils and macrophages are promptly recruited and rapidly accumulate at the site of injury^1^. These cells combat infection and also serve as a source of various pro-healing growth factors and cytokines (VEGF, IL-1, TNF, PDGF)^8,43^ As such, their depletion in DFUs contributes to inhibition of healing. Indeed, neutrophil and macrophage-recruiting cytokines such as IL-6, CXCL1, and CXCL8 are downregulated in DFU. Our data further support the notion of impaired quality of immune-cell response in DFUs, as several genes involved in immune-cell activation and function (i.e., CCL8, CSF3) were downregulated in our datasets, consistent with previous descriptions of the DFU phenotype^17,28^.

Our analysis showed mast cells to be present in oral, skin, and DFUs. Studies have shown that deficiency of mast cells delayed wound healing in mouse models of diabetes^58^. Mast cells are sources of several factors involved in the wound-healing process and interact with various cell types including macrophages, endothelial cells and eosinophils^59,60^. We found eosinophils to be also present in DFUs. Cross-talk between eosinophils and mast cells have been demonstrated in several allergic disorders that include asthma, allergic hypersensitivity and atopic dermatitis^60,61^. While the role of eosinophils and their cross-talk with mast cells during wound healing are poorly understood, our data demonstrate that the presence of eosinophils specific for DFUs may have a role in contributing to the wound chronicity. Furthermore, *Staphylococcus aureus* the most prevalent pathogen in both DFU^62^ and atopic dermatitis^63^ has shown to be responsible for increased recruitment of mast cells and eosinophils in animal models of atopic dermatitis^64^, suggesting similar traits in DFUs.

Overall, we present a comprehensive comparative analysis of the transcriptional networks underlying the pathogenesis of DFUs. Our data demonstrate that impaired activation, proliferation, and survival of immune cells in the DFU environment contribute to a downregulated inflammatory response. Downregulation of FOXM1 contributes to decreased immune cell infiltrate and diminished inflammatory response in diabetic wounds. The identification of pathways responsible for downregulation of inflammation and delayed wound healing can be exploited in the clinical setting for diagnostic and prognostic purposes. Ultimately, these findings have significant clinical implications in the development of novel therapeutic avenues.

## Methods

### Patient demographics and tissue collection

Full-thickness DFU (*n* = 13, mean age ± standard deviation = 56 ± 13, 13 males) and DFS (*n* = 8, mean age ± standard deviation = 66 ± 13, 7 males, 1 female) samples were obtained from patients receiving standard care at the University of Miami Hospital Wound Clinic, as previously described^6,27,65^. The protocols including written informed consent were approved by the university Institutional Review Board (IRB #20140473; #20090709). Inclusion criteria for DFU were (1) diabetes mellitus; (2) an ulcer on the plantar aspect of their foot that is larger than 0.5 cm^2^; (3) neuropathy; (4) age 21 years or older; (5) wound duration >4 weeks; (6) hemoglobin A1c: ≤13.0%. Ulcers with clinical signs of infection were excluded. Exclusion criteria for DFU were (1) active cellulitis; (2) osteomyelitis; (3) gangrene; (4) vascular insufficiency (defined as an ankle-brachial index (ABI) <0.7 and for those with an ABI >1.3; (5) revascularization to the ipsilateral lower extremity in the last 6 weeks; (6) any experimental drugs taken or applied topically to the wound for 4 weeks preceding the study. Biopsies were stored in RNALater (Applied Biosystems) for RNA isolation or fixed in formalin (paraffin embedding). Oral and skin wounds were previously described^23^. Due to limited size of human tissue samples majority of material was utilized for the genomic and tissue analyses and are no longer available.

### Mice

All animal studies were carried out according to the protocol approved by the Animal and Care Committee at the National Institute of Arthritis and Musculoskeletal and Skin Diseases (protocol # A019-02-01) and by the University of Miami Institutional Animal Care and Use Committee (protocol # 18–053). CD1 mice were purchased from Charles River, USA (Strain number: 022). Db/db mice were purchased from Jackson Laboratory. Both male and female mice were used in the studies. Treatments were performed at 7–9 weeks of age and all experiments were conducted using littermate controls.

### Induction of diabetic mouse model

CD1 mice were induced to be diabetic by multiple low-dose injections of streptozotocin (STZ) at 4–5 weeks old. Mice were fasted for 6 h and then injected with vehicle or STZ (intraperitoneal injection, 60 mg/kg dissolved in 50 mM sodium citrate buffer, pH 4.5) for 5 consecutive days. Fasting blood glucose levels were measured 1 week after STZ injections. Mice with fasting blood glucose levels >300 mg/dl were considered diabetic. Duration of diabetes was maintained for 6 weeks to allow for pathophysiological effects of diabetes to occur and diabetic mice received low-dose subcutaneous insulin injections (0.5 U/30 µl; Lantus) every other day to maintain viability of the mice. After 6 weeks, mice were used for wound-healing assays.

### Wound-healing assay in vivo

Full-thickness wounds were created as previously described^23,26^. At minimum three mice were randomized to each of the experimental groups. Briefly, mice were anesthetized, and hair was shaved on dorsal skin and cleaned with 70% ethanol. Two excisional wounds per each mouse were created using 6 mm full-thickness sterile biopsy punch (Integra Miltex, York, PA) and treated with either 150 µM FDI-6 (Sigma-Aldrich; SML1392) or vehicle (DMSO) dissolved in 1x sterile PBS and covered with film dressing (PERME-ROLL; NITTO DENKO, Osaka, Japan). Treatments were applied every 2 days and digitally photographed at indicated time points and wound areas were measured using Fiji. Changes in wound area are expressed as percentages of initial wound area.

### RNA sequencing and bioinformatics analysis

Preparation and sequencing of RNA libraries for patient DFUs was carried out in the John P. Hussman Institute for Human Genomics Center for Genome Technology. Briefly, total RNA was used as input for the RiboZero Transcriptome Directional RNAseq sample prep to create ribosomal RNA depleted libraries. Each sample was sequenced to 40 million raw reads in a single end 75 bp sequencing run on the Illumina NextSeq500 and then demultiplexed and converted to FastQ using bcl2fastq 2.17.1. FastQ files were moved to the NIAMS Biodata Mining and Discovery Section for analysis. Reads were mapped to human genome build hg19 using TopHat 2.1.1. Gene expression values (RPKM, reads per kilobase exon per million mapped reads) were calculated and log2 transformed (with a 0.1 offset), ANOVA performed to find differentially expressed genes (DEG), heatmap and volcano plots created with Partek Genomic Suites 7.18.0723. Pathway analysis was conducted using IPA (Qiagen: www.ingenuity.com). IPA software used Fishers exact test to detect significantly enriched pathways and biological processes with *P* value of 0.05 or less were considered significant.

### Immunohistochemistry

Paraffin embedded tissue sections of discarded DFUs, foot skin (FS), oral and skin wounds were used for staining with anti-phospho-STAT3 (1:100; Abcam), anti-MPO (1:1500; Abcam), anti-CD68 (1:800; Abcam), anti-PCNA (1:1000; Cell Signaling), anti-Keratin 5 (1:1000; LSBio), anti-FOXM1 (1:600; Cell Signaling), anti-STAT3 (1:100; Cell Signaling), anti-TNFα (1:25; Abcam), and anti-Ki67 (1:200; Abcam). Murine wounds were excised at day 4 post-wounding and fixed in 4% paraformaldehyde overnight at 4 °C and sections were used for staining with anti-MPO (1:1500; Abcam) and anti-Keratin 5 (1:1000; LSBio). Stainings were visualized with either Alexa Fluor 488-conjugated goat anti-rabbit antibody (1:300; Invitrogen), Alexa Fluor 555-conjugated goat anti-guinea pig antibody (1:300; Invitrogen), Alexa Fluor 647-conjugated goat anti-mouse antibody (1:300; Invitrogen), and mounted with VECTASHIELD antifade mounting media with DAPI (Vectorlabs) to visualize cell nuclei. Specimens were analyzed using a Zeiss LSM 780 confocal microscope and images were acquired with Zen software (Carl Zeiss).

### Tissue processing and flow cytometric analysis

Wound edge skin at specific time points was collected and full-thickness skin samples were minced with surgical scissors and incubated for 30 min in dispase I solution (Roche, Basel, Switzerland) (2.4 μg/mL) at 37 °C. This was followed by incubation at 37 °C for 3 h with 2 mg/ml Collagenase D (Roche) at 37 °C under constant agitation. Obtained single-cell suspensions were washed with IMEM (Gibco-Thermo Fisher Scientific) supplemented with 10% heat-inactivated FBS, 2 mM L-glutamine, 0.15% sodium hydrogencarbonate, 1 mM sodium pyruvate, nonessential amino acids, and 50 μg/ml gentamycin. Single-cell suspensions were incubated with combinations of fluorophore-conjugated antibodies against the following surface markers: CD45-APCCy7 (30-F11), CD3e-BV711(17A2), F4/80-PE (BM8), CD11b-PE Dazzle 594 (M1/70), Ly6C-PerCP (HK1.4), Ly6G-BUV395 (1A8) in Hank’s buffered salt solution (HBSS) for 30 min at 4 °C and then washed. LIVE/DEAD Fixable Violet Dead Cell Stain Kit (Invitrogen Life Technologies) was used to exclude dead cells. Cells were then fixed for 30 min at 4 °C using BD Cytofix/Cytoperm (Becton Dickinson) and washed twice. All antibodies were purchased from Biolegend and BD Biosciences. Cell acquisition was performed on a BD LSR-Fortessa–HTS flow cytometer using FACSDiVa software (BD Biosciences) and data were analyzed using FlowJo software (TreeStar).

### Real-time reverse transcriptase PCR

RNA was reverse transcribed using a qScript cDNA kit (QuantaBio, Beverly, MA) and real-time PCR was performed in triplicates using the Bio-Rad CFX Connect thermal cycler and detection system and a PerfeCTa SYBR Green Supermix (QuantaBio, Beverly, MA). Relative expression was normalized for levels of ARPC2. The primer sequences for ARPC2 are forward primer (5′-TCCGGGACTACCTGCACTAC-3′) and reverse primer (5′-GGTTCAGCACCTTGAGGAAG-3′); STAT3 forward primer (5′-CAGCAGCTTGACACACGGTA-3′) and reverse primer (5′-AAACACCAAAGTGGCATGTGA-3′); FOXM1 forward primer (5′-CGTCGGCCACTGATTCTCAAA-3′) and reverse primer (5′-GGCAGGGGATCTCTTAGGTTC-3′); TNFα forward primer (5′-CCGAGGCAGTCAGATCATCTT-3′) and reverse primer (5′-AGCTGCCCCTCAGCTTGA-3′). Statistical comparisons were performed using two-way ANOVA.

### Statistical analysis

Pathway enrichment statistics were calculated within the Ingenuity software package using Fisher’s exact test with Benjamini–Hochberg correction for multiple testing. Upstream regulators and gene ontology enrichment *P*-values were similarly calculated within IPA using Fisher’s exact test. Statistics for qPCR validation studies were performed using two-way ANOVA followed by Tukey’s post hoc test. Error bars for statistical analyses were defined as mean ± standard deviation (S.D.) or standard error of the mean (S.E.M.) where appropriate.

### Reporting summary

Further information on research design is available in the Nature Research Reporting Summary linked to this article.

## Supplementary information

Supplementary Information

Reporting Summary

## Data Availability

The authors declare that all data supporting the findings of this study are available within the article and its supplementary information files or from the corresponding author upon reasonable request. Raw data and analyzed RNA-seq data supporting the findings in this study have been deposited in the GEO database under accession code: GSE134431. Raw and analyzed RNA-seq data regarding oral and skin acute human wounds have been deposited previously in the Gene Expression Omnibus (GEO) database under accession codes: (GSE97615, GSE97616, GSE97617), as previously described^23^. Source data are provided with this paper.

## Source data

Source Data
