# Supplementary material for: Deregulated immune cell recruitment orchestrated by FOXM1 impairs human diabetic wound healing
Source: Nat Commun. 2020 Sep 16;11:4678. doi: 10.1038/s41467-020-18276-0 (PMC7495445; doi:10.1038/s41467-020-18276-0)
Supplement: Supplementary file 1 — Supplementary Information [file 41467_2020_18276_MOESM1_ESM.pdf]

SUPPLEMENTAL MATERIAL

Deregulated immune cell recruitment orchestrated by FOXM-1 impairs human diabetic wound healing

Andrew P. Sawaya, Rivka C. Stone, Stephen R. Brooks, Irena Pastar, Ivan Jozic, Kowser Hasneen, Katelyn O'Neill, Spencer Mehdizadeh, Cheyanne R. Head, Natasa Strbo, Maria I. Morasso and Marjana Tomic-Canic

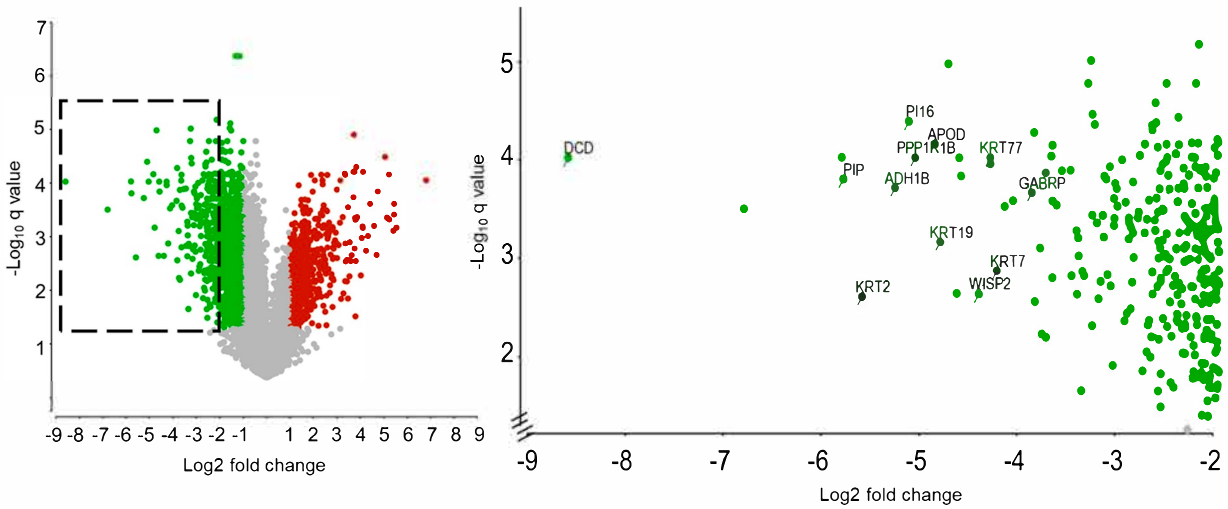

Supplementary Figure 1. Volcano plot of most significantly downregulated genes in DFUs. Volcano plot indicating differentially regulated genes in DFUs. Dotted line region is magnified on the right panel highlighting some of the most significantly down-regulated genes in DFUs

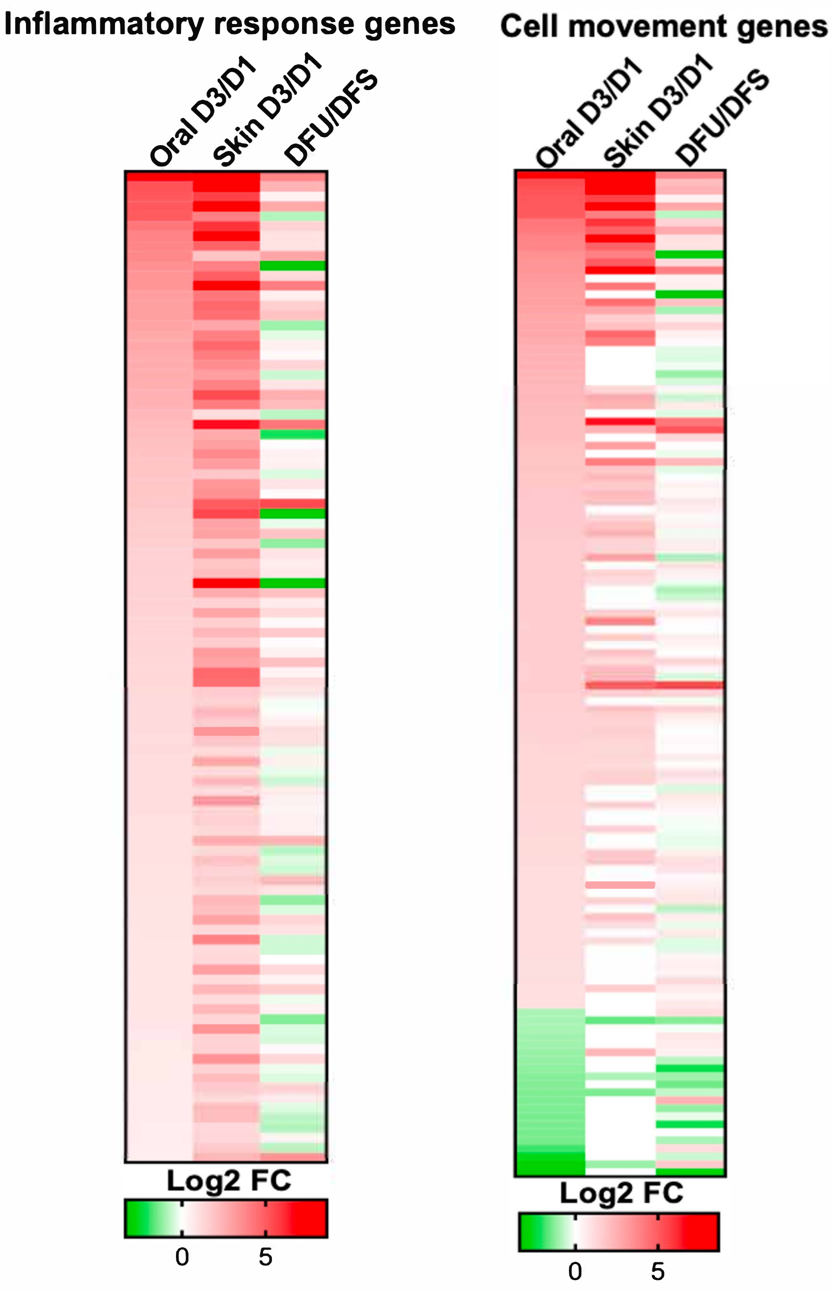

Supplementary Figure 2. Downregulation of genes in DFUs involved in inflammatory response and cellular movement processes.

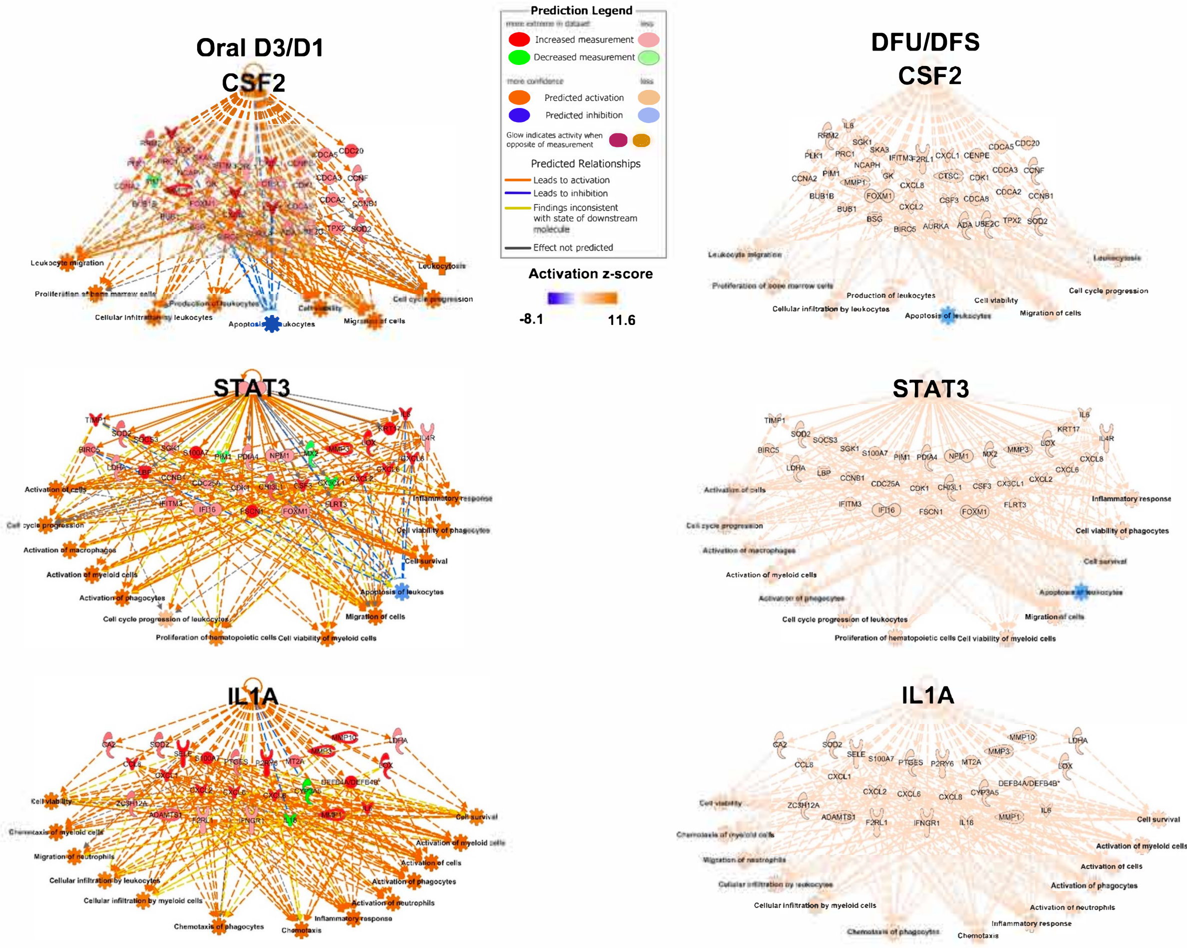

Supplementary Figure 3. Upstream regulators CSF2, STAT3 and IL1A partially activated in DFUs compared to oral and skin wounds.

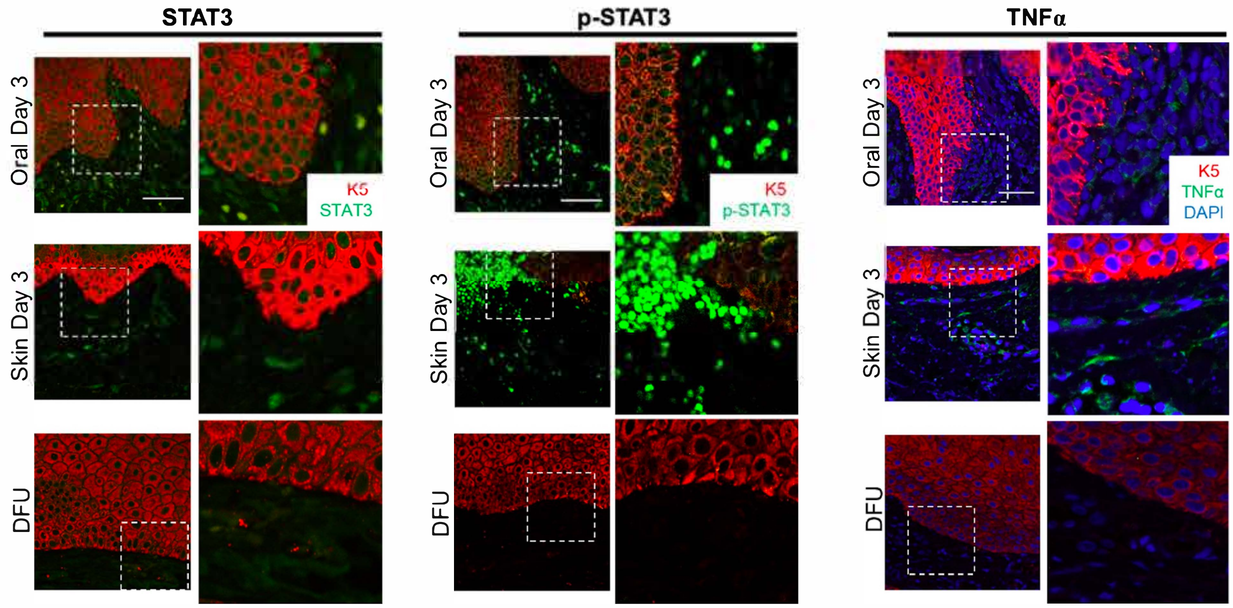

Supplementary Figure 4. Validation of upstream regulators STAT3, p-STAT3 and TNFα in DFUs compared to oral and skin wounds. Representative pictures of oral day 3 wounds, skin day 3 wounds and DFUs to show basal marker K5, and upstream regulators that are downregulated in DFUs compared to oral and skin wounds. Experiments were performed once with 3 biologically independent patient samples per group with minimum duplicate technical replicates. Scale bar= 50 µm, magnification 20x.

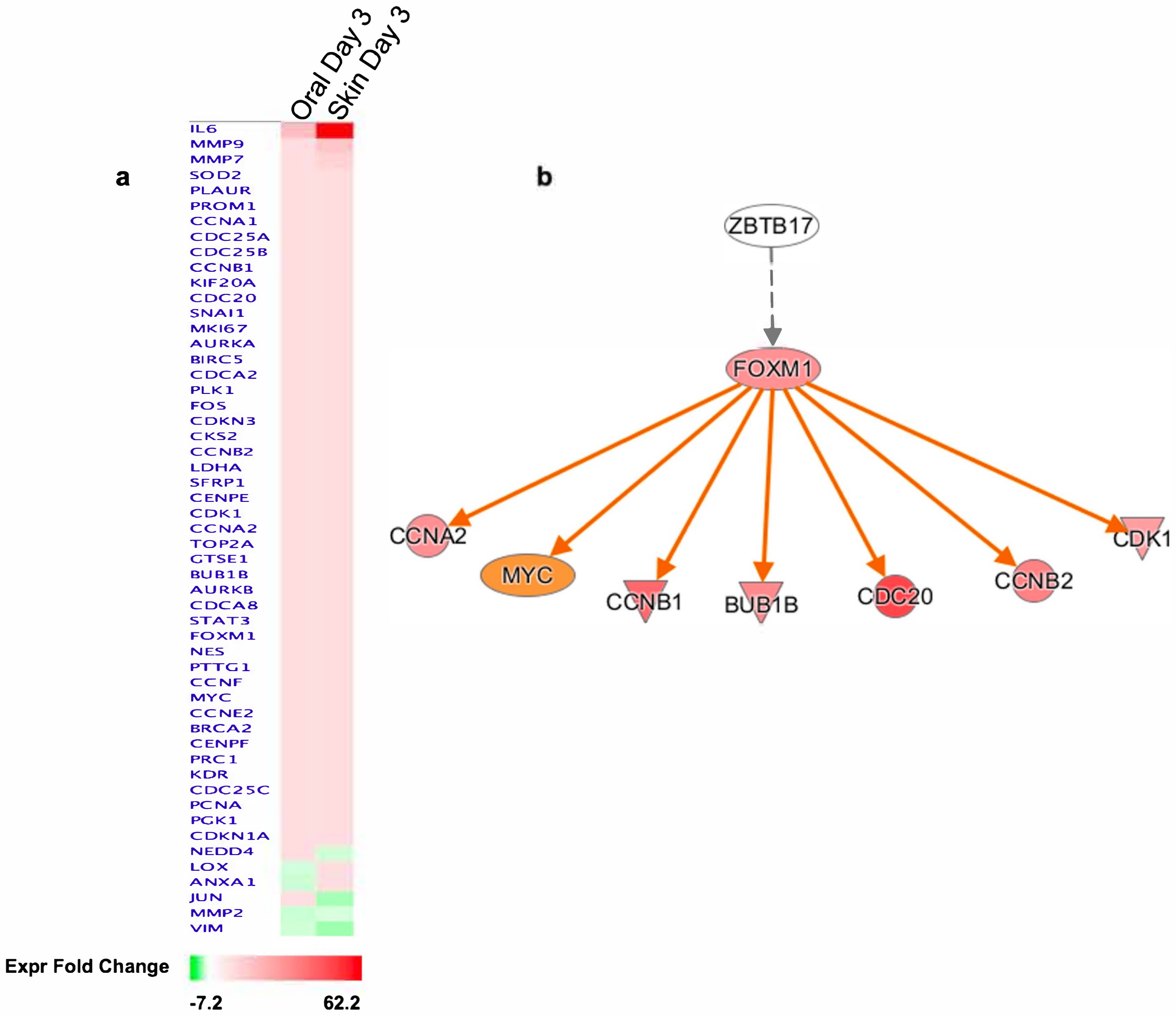

Supplementary Figure 5. FOXM1 pathway activation in human acute oral and skin wounds. a. Heat-map of genes involved in FOXM1 pathway demonstrating oral and skin wounds share 50/54 genes commonly regulated. b. Upstream regulator, ZBTB17, that acts as an upstream negative regulator of FOXM1 pathway is suppressed in oral and skin wounds.

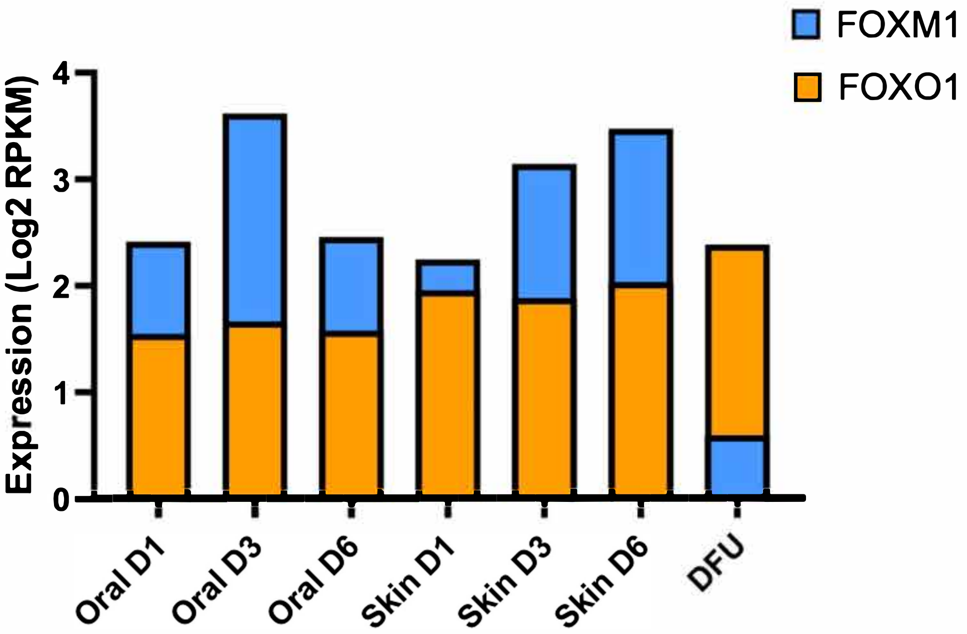

Supplementary Figure 6. Time course of FOXM1 and FOXO1 expression during wound healing in oral and skin wounds compared to DFUs. FOXM1 is induced upon wounding in oral and skin day 3 wounds compared to suppression in DFUs. FOXO1 expression does not change during wounding in oral and skin wounds and is comparable to DFUs. RPKM= Reads per kilobase of transcript per million mapped reads.

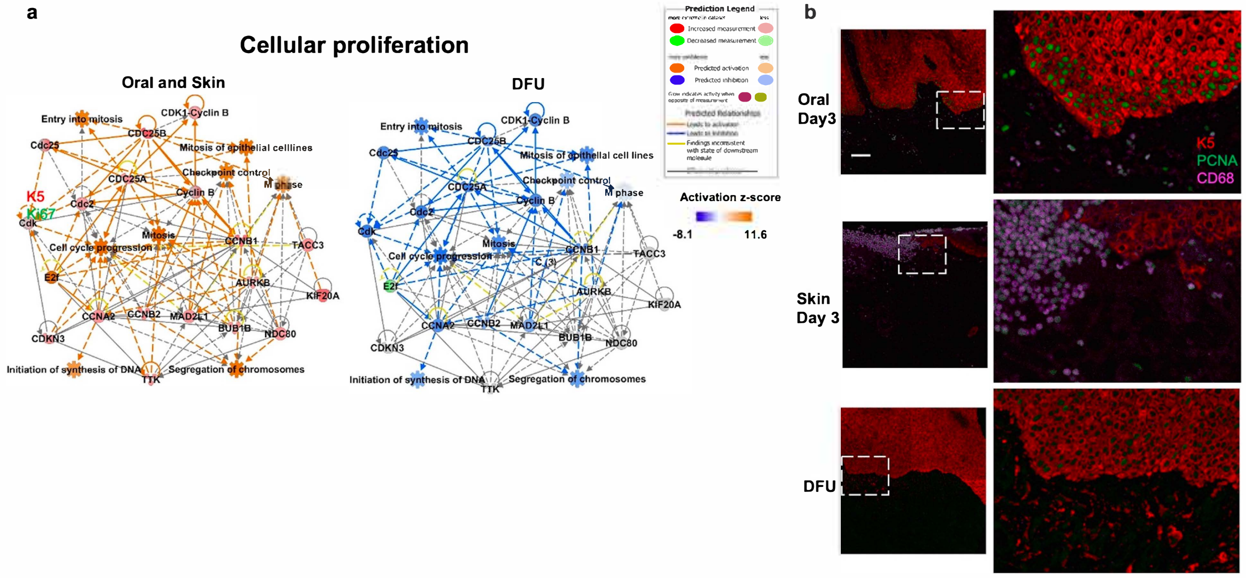

Supplementary Figure 7. Inhibition of proliferation in DFUs. a IPA-predicted network of cellular proliferation from oral and skin specific genes compared to DFU specific genes. b Representative pictures of oral day 3 wounds, skin day 3 wounds and DFUs to show basal marker K5, proliferation marker PCNA and macrophage marker CD68. Decreased PCNA and CD68 demonstrates decreased proliferation of macrophages in DFUs compared to oral and skin wounds. Experiments were performed once with 3 biologically independent patient samples per group with minimum duplicate replicates. Scale bar= 100 μm.

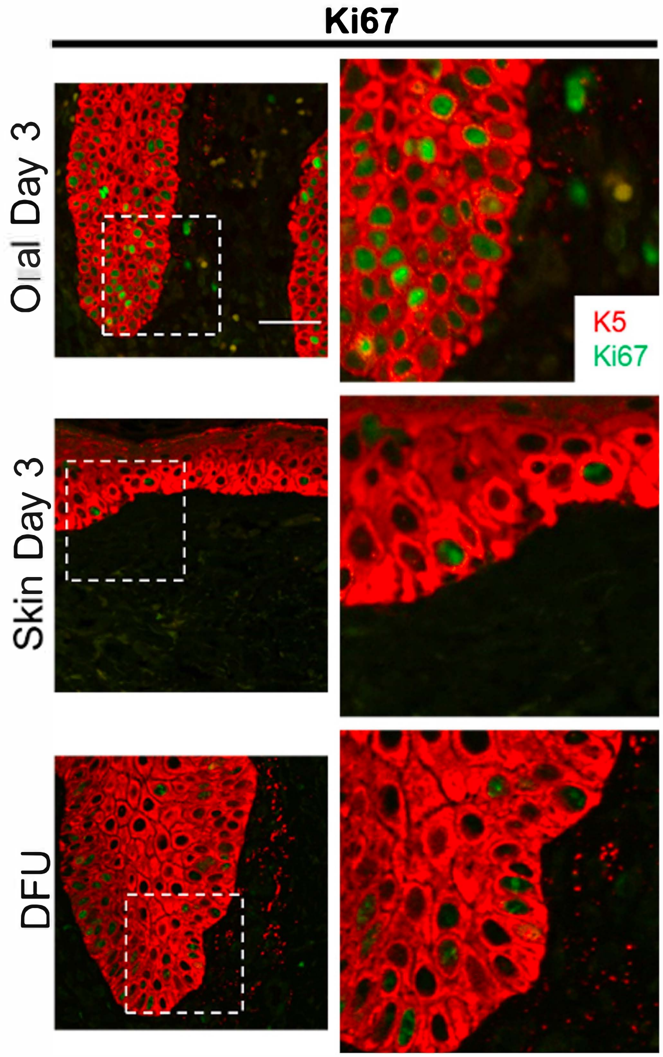

Supplementary Figure 8. Hyperproliferation in epidermis of oral, skin and DFU wounds. Representative pictures of oral day 3 wounds, skin day 3 wounds and DFUs to show basal marker K5 and proliferation marker Ki67. Ki67 staining demonstrates increased epidermal proliferation of keratinocytes in oral, skin and DFU wounds. Experiments were performed once with 3 biologically independent patient samples per group with minimum duplicate replicates. Scale bar= 50 µm, magnification 20x.

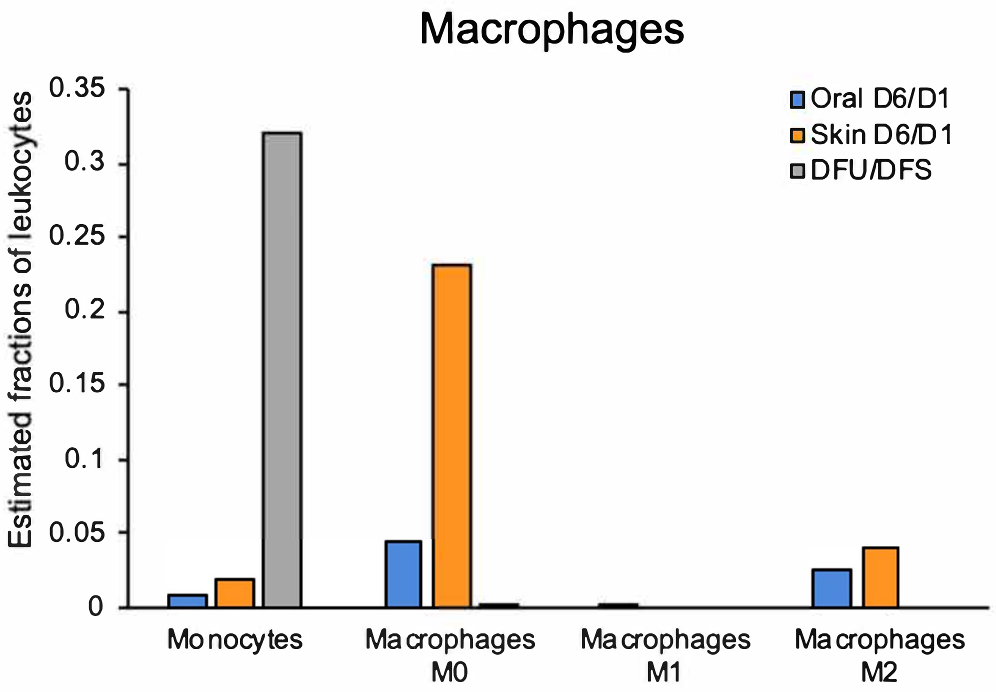

Supplementary Figure 9. Prediction of macrophages populations in oral and skin day 6 wounds compared to DFUs. Estimated proportions of macrophages in oral and skin wounds at day 6 post wounding shows M2 macrophages to be present.

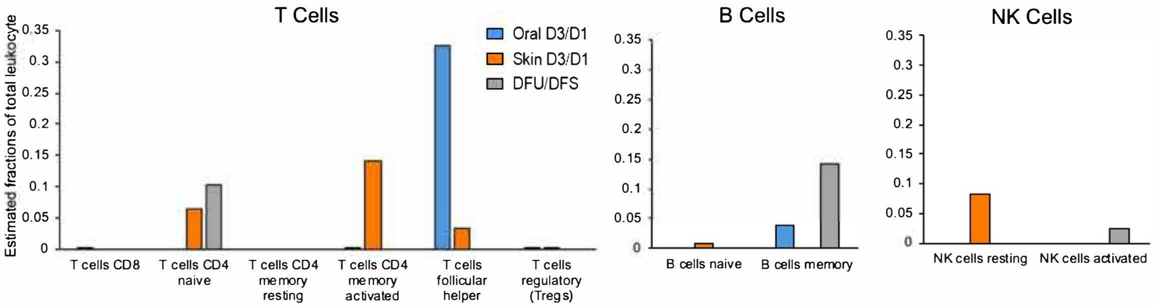

Supplementary Figure 10. Prediction of immune cell infiltrates in oral, skin day 3 wounds and DFUs. Estimated proportions of different subsets of leukocytes (T cells, B cells and NK cells) in DFUs compared to oral and skin wounds.

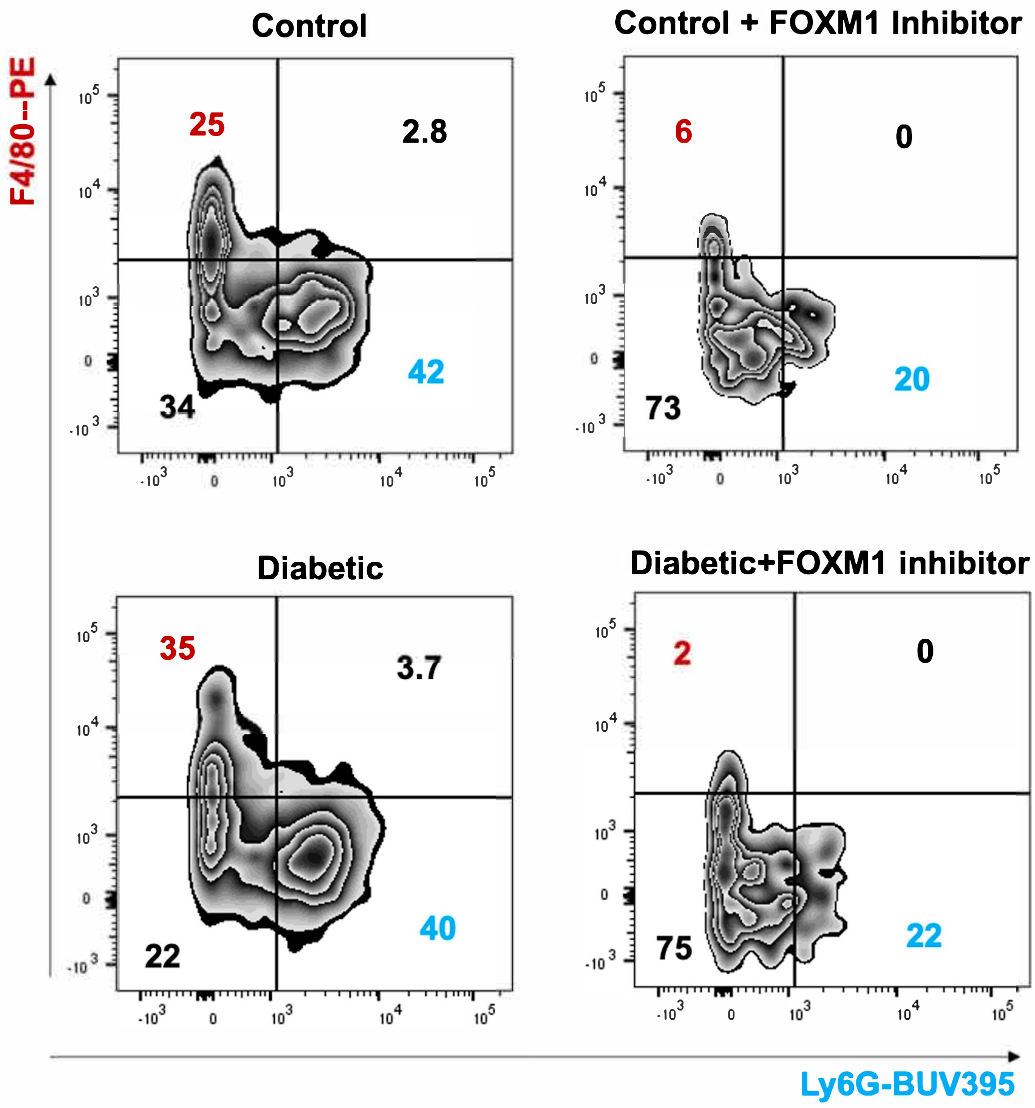

Supplementary Figure 11. Inhibition of FOXM1 decreases frequency of macrophages and neutrophils in the wounds of STZ-induced diabetic mice. Representative zebra plots of F4/80 and Ly6G expression on gated CD11b+ cells from wounded skin after topical treatment with either vehicle or FOXM1 inhibitor FDI-6 on day 4. Numbers in representative plots indicate percent positive cells in each quadrant. Treatment of wounds with FDI-6 resulted in decreased macrophages and neutrophils compared to vehicle treated wounds.

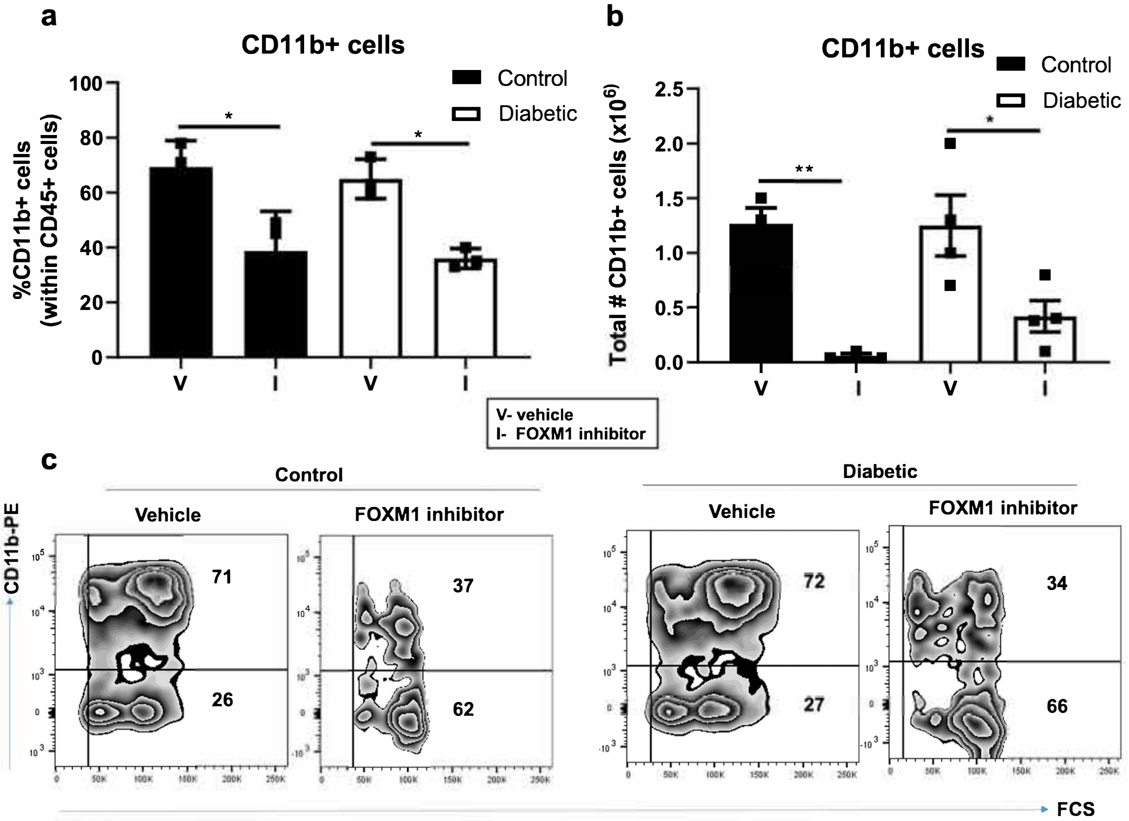

Supplementary Figure 12. Inhibition of FOXM1 decreases frequency of myeloid, CD11b+ cells, in diabetic wounds. a-c Diabetic (STZ-induced) and CD-1 control mice were wounded and treated topically with either the FOXM1 inhibitor FDI-6 or vehicle. Wound edge skin at day 4 was collected and frequencies of total numbers of myeloid, CD11b+ cells was determined by flow cytometry. Data represent three mice per group. Data presented as mean ± SEM. (a) *P =0.018 for control group and *P=0.012 for diabetic group, (b) **P =0.009 for control group and *P=0.049 for diabetic group as calculated using one-way ANOVA with Tukey's multiple comparisons test (a,b). Source data are provided as a Source Data file. c Representative zebra plots of CD11b expression on gated CD45+ single cells from wounded skin after topical treatment with either vehicle or FOXM1 inhibitor FDI-6 on day 4. Numbers in representative plots indicate percent positive cells in each quadrant. Treatment of wounds with FDI-6 resulted in decreased CD11b+ cells compared to vehicle treated wounds.

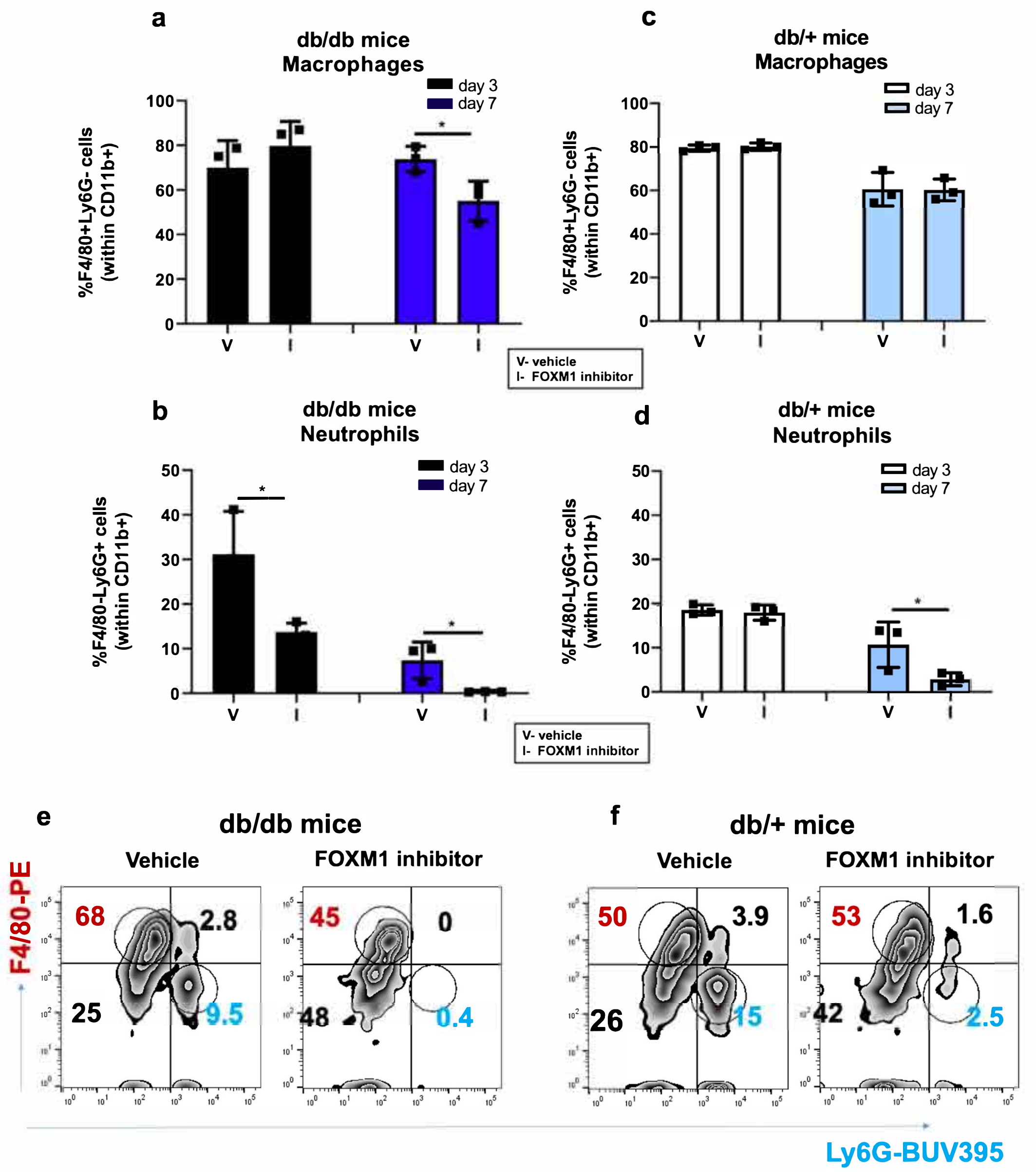

Supplementary Figure 13. Inhibition of FOXM1 decreases frequency of macrophages and neutrophils in db/db and db/+ wounds. a-d db/db (a,b) and db/+ (c,d) mice were wounded and treated topically with either the FOXM1 inhibitor FDI-6 or vehicle every other day for 7 days. Wound edge skin at day 3 and 7 was collected and frequencies of macrophages (F4/80+Ly6G-) and neutrophils (F4/80-Ly6G+) within gated myeloid cells, was determined by flow cytometry. Data represent three mice per group. Data presented as mean ± SEM. Source data are provided as a Source Data file. (a) *P= 0.036, (b) *P=0.039 for day 3 group and *P=0.037 for day 7 group, (d) *P=0.03, as calculated using two-tailed unpaired Student t test (a-d). e-f Representative zebra plots of F4/80 and Ly6G expression on gated CD11b+ cells from wounded skin after topical treatment with either vehicle or FOXM1 inhibitor FDI-6 on day 7. Numbers in representative plots indicate percent positive cells in each quadrant. Treatment of wounds with FDI-6 resulted in decreased macrophages and neutrophils compared to vehicle treated wounds.

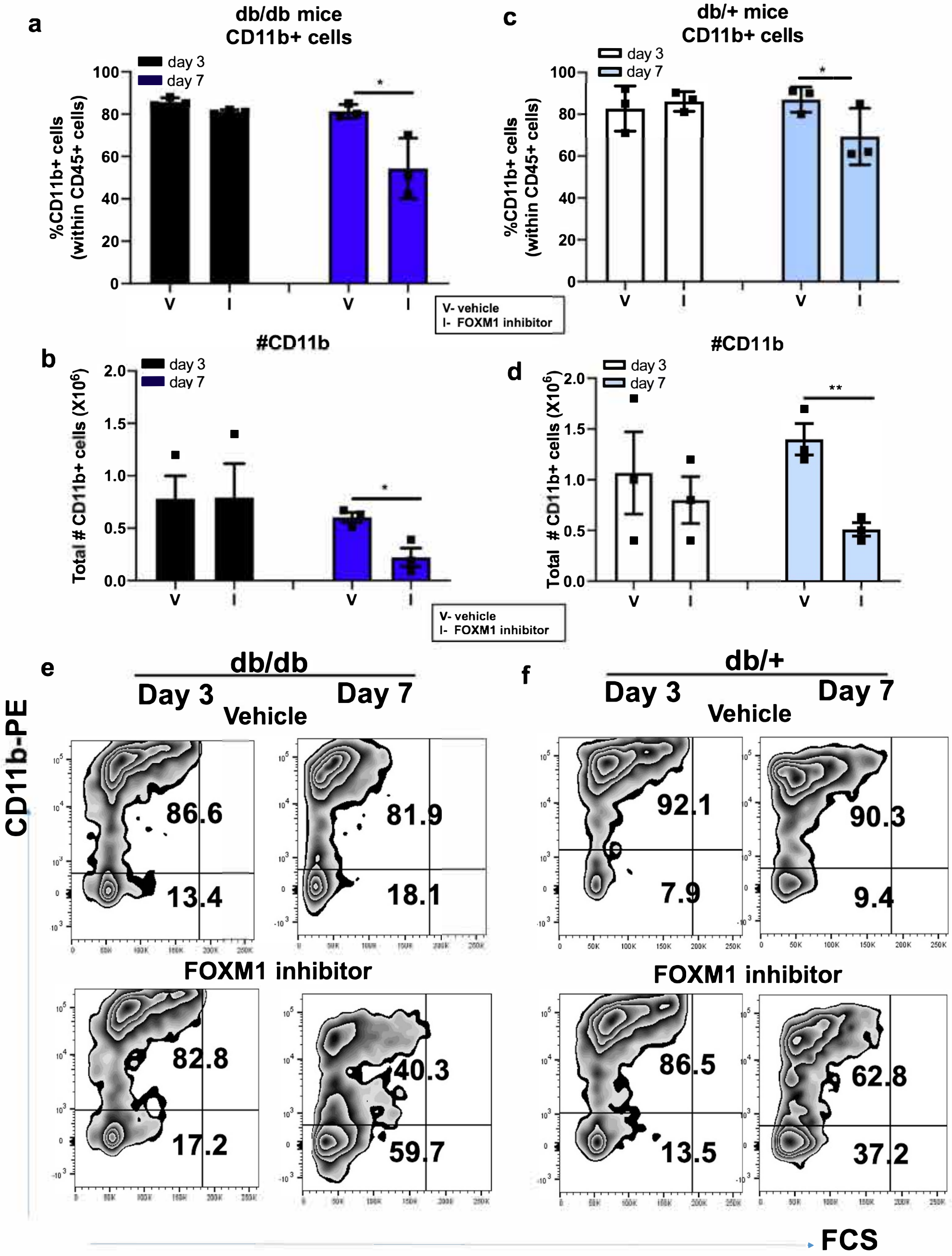

Supplementary Figure 14. Inhibition of FOXM1 decreases frequency of myeloid, CD11b+ cells, in db/db and db/+ wounds. a-d db/db (a,b) and db/+ (c,d) mice were wounded and treated topically with either the FOXM1 inhibitor FDI-6 or vehicle every other day for 7 days. Wound edge skin at day 3 and 7 were collected and frequencies of myeloid, CD11b+ cells was determined by flow cytometry. Data represent three mice per group. Data presented as mean ± SEM. Source data are provided as a Source Data file. (a) *P=0.033, (b)*P =0.019, (c) P=0.045, (d) **P=0.006 as calculated using two-tailed Student t test (a-d). e-f Representative zebra plots of CD11b expression on gated CD45+ single cells from wounded skin after topical treatment with either vehicle or FOXM1 inhibitor FDI-6 on day 3 and 7. Numbers in representative plots indicate percent positive cells in each quadrant. Treatment of wounds with FDI-6 resulted in decreased CD11b+ cells compared to vehicle treated wounds.

FOXM1 interacting factors involved in regulating wound healing that are suppressed in DFUs

FOXM1- wound healing-interactions	Expression in acute wounds (Oral and Skin)	Expression in DFU	Evidence supporting promotion of healing
IL6	Increased	No change	*IL6*$^{-/-}$ mice exhibit impaired healing in part due to delayed macrophage infiltration and neutrophil trafficking[1,2]
MMP9	Increased	No change	FOXM1 regulates MM9 thru JNK to stimulate cell migration[3]
SOD2	Increased	Decreased	SOD2 is present in mitochondria of immune cells, in particular neutrophils, promoting wounding-triggered intracellular killing of microorganisms via generation of reactive oxygen species (ROS)[4]

Supplementary Table 1. FOXM1 interacting proteins involved in stimulating wound healing downregulated in DFUs compared to oral and skin acute wounds.

Supplementary References

1. Gallucci RM, et al. Impaired cutaneous wound healing in interleukin-6-deficient and immunosuppressed mice. FASEB journal : official publication of the Federation of American Societies for Experimental Biology 14, 2525-2531 (2000).

2. McFarland-Mancini MM, et al. Differences in wound healing in mice with deficiency of IL-6 versus IL-6 receptor. J Immunol 184, 7219-7228 (2010).

3. Wang IC, et al. FoxM1 regulates transcription of JNK1 to promote the G1/S transition and tumor cell invasiveness. The Journal of biological chemistry 283, 20770-20778 (2008).

4. Bellot GL, et al. MnSOD is implicated in accelerated wound healing upon Negative Pressure Wound Therapy (NPWT): A case in point for MnSOD mimetics as adjuvants for wound management. Redox biology 20, 307-320 (2019).
