# Supplementary material for: Deregulated immune cell recruitment orchestrated by FOXM1 impairs human diabetic wound healing
Source: Nat Commun. 2020 Sep 16;11:4678. doi: 10.1038/s41467-020-18276-0 (PMC7495445; doi:10.1038/s41467-020-18276-0)
Supplement: Supplementary file 2 — Reporting Summary [file 41467_2020_18276_MOESM2_ESM.pdf]

nature research

Corresponding author(s): Marjana Tomic-Canic and Maria I. Morasso

Last updated by author(s): July 15, 2020

Reporting Summary

Nature Research wishes to improve the reproducibility of the work that we publish. This form provides structure for consistency and transparency in reporting. For further information on Nature Research policies, see our Editorial Policies and the Editorial Policy Checklist.

Statistics

For all statistical analyses, confirm that the following items are present in the figure legend, table legend, main text, or Methods section.

n/a	Confirmed	
☐	☒	The exact sample size (n) for each experimental group/condition, given as a discrete number and unit of measurement
☐	☒	A statement on whether measurements were taken from distinct samples or whether the same sample was measured repeatedly
☐	☒	The statistical test(s) used AND whether they are one- or two-sided<br>*Only common tests should be described solely by name; describe more complex techniques in the Methods section.*
☒	☐	A description of all covariates tested
☒	☐	A description of any assumptions or corrections, such as tests of normality and adjustment for multiple comparisons
☐	☒	A full description of the statistical parameters including central tendency (e.g. means) or other basic estimates (e.g. regression coefficient) AND variation (e.g. standard deviation) or associated estimates of uncertainty (e.g. confidence intervals)
☐	☒	For null hypothesis testing, the test statistic (e.g. F, t, r) with confidence intervals, effect sizes, degrees of freedom and P value noted<br>*Give P values as exact values whenever suitable.*
☒	☐	For Bayesian analysis, information on the choice of priors and Markov chain Monte Carlo settings
☒	☐	For hierarchical and complex designs, identification of the appropriate level for tests and full reporting of outcomes
☒	☐	Estimates of effect sizes (e.g. Cohen's d, Pearson's r), indicating how they were calculated

Our web collection on statistics for biologists contains articles on many of the points above.

Software and code

Policy information about availability of computer code

Data collection	No software was used to collect the data.
Data analysis	IPA software Summer 2019 version<br>BD FACSDiva version v8.0.1<br>Fiji for Mac OSX 2019<br>Partek Genomics Suite software, version 7.0<br>ImageJ version 1.52p

For manuscripts utilizing custom algorithms or software that are central to the research but not yet described in published literature, software must be made available to editors and reviewers. We strongly encourage code deposition in a community repository (e.g. GitHub). See the Nature Research guidelines for submitting code & software for further information.

Data

Policy information about availability of data

All manuscripts must include a data availability statement. This statement should provide the following information, where applicable:

- Accession codes, unique identifiers, or web links for publicly available datasets
- A list of figures that have associated raw data
- A description of any restrictions on data availability

Details are provided in data availability statement in the methods section of the manuscript. Raw and analyzed RNA-Seq data have been deposited in the Gene Expression Omnibus (GEO) site GSE97615, GSE97616, GSE97617. Data from GSE134431 will be publicly available via dbGap. Manuscript text has been updated accordingly. Figures 1-5 are associated with these datasets.

Field-specific reporting

Please select the one below that is the best fit for your research. If you are not sure, read the appropriate sections before making your selection.

- [x] Life sciences
- [ ] Behavioural & social sciences
- [ ] Ecological, evolutionary & environmental sciences

For a reference copy of the document with all sections, see nature.com/documents/nr-reporting-summary-flat.pdf

Life sciences study design

All studies must disclose on these points even when the disclosure is negative.

Sample size	Full thickness DFU (n=13) and foot skin (n=8) samples were obtained from patients receiving standard care at the University of Miami Hospital Wound Clinic. Sample size was determined based on quality assessments as described in Exp Dermatol. 2013 Mar;22(3):216-8. doi: 10.1111/exd.12104. For the mouse experiments at minimum three mice (with two wounds each) were used for each experimental groups. No sample size calculations were used.
Data exclusions	No data exclusions.
Replication	All reported findings results from multiple replicated experiments as indicated in Results and/or Figure legends.
Randomization	As the collection of human samples for this study was retrospective, with predefined designation of DFU vs DFS tissue, randomization was not relevant. Mice within biological group (WT, STZ-induced diabetes and db/db) were randomized to vehicle control and treatment groups.
Blinding	Blinding for FACS analyses was performed, and blinding for the human analyses was not possible, due to recognizable morphology of the specific tissue (oral, skin and DFU). Blinding for the animal studies (db/db and streptozocin mice) was not feasible due to the sensitivity of the wounding procedure. The same investigator prepared the treatment solutions performed the wounding.

Reporting for specific materials, systems and methods

We require information from authors about some types of materials, experimental systems and methods used in many studies. Here, indicate whether each material, system or method listed is relevant to your study. If you are not sure if a list item applies to your research, read the appropriate section before selecting a response.

Materials & experimental systems

n/a	Involved in the study	
☐	☒	Antibodies
☒	☐	Eukaryotic cell lines
☒	☐	Palaeontology and archaeology
☐	☒	Animals and other organisms
☐	☒	Human research participants
☒	☐	Clinical data
☒	☐	Dual use research of concern

Methods

n/a	Involved in the study	
☒	☐	ChIP-seq
☐	☒	Flow cytometry
☒	☐	MRI-based neuroimaging

Antibodies

Antibodies used	Anti phospho-STAT3 (1:100; Abcam catalog number: ab76315, lot number: GR287047-42), anti MPO (1:1500; Abcam, catalog number: ab9535, lot number:GR3177797-5), anti CD68 (1:800; Abcam, catalog number: ab955, lot number:GR3250836-3), antiPCNA (1:1000; Cell Signaling, catalog number: 13110S, lot number: 4), anti Keratin 5 (1:1000; LSBio, catalog number:LS-c22715-100), anti FOXM1 (1:600; Cell Signaling, catalog number: 20459S, lot number:1), anti STAT3 (1:100; Cell Signaling, catalog number: 12640S, lot number:4), anti TNF! (1:25; abcam, catalog number: ab1793, lot number: GR3313040-1), anti Ki67 (1:200; abcam, catalog number:ab15580, lot number:GR3220920-1), anti CD45-APCCy7 (Biolegend, clone: 30-F11, catalog number:557659, lot number: 7088545), anti CD3e-BV711(Biolegend, clone:17A2, catalog number: 100241, lot number:B296084), anti F4/80-PE (Biolegend, clone:BM8, catalog number:123110, lot number:B281021), anti CD11b-PE Dazzle 594 (Biolegend, clone:M1/70, catalog number:101256, lot number:B211425), anti Ly6C-BV605 (Biolegend, clone:HK1.4, catalog number:128036, lot number:B266313), Ly6G- BUV395(BD Biosciences, clone:1A8, catalog number:565964, lot number:9346020), Alexa Fluor 488-conjugated goat anti-rabbit antibody (1:300; Invitrogen, catalog number: A11008, lot number:1981125), Alexa Fluor 555-conjugated goat anti-guinea pig antibody (1:300; Invitrogen, catalog number: A21435, lot number:1932510), Alexa Fluor 647-conjugated goat anti-mouse antibody (1:300; Invitrogen, catalog number: A21235, lot number: 564521).
Validation	Each antibody came with a validation of specificity from the respective manufacturer. Anti phospho-STAT3 was deemed suitable for: WB, IP, IHC-P, Dot blot, ICC/IF and reacts with: Mouse, Rat, Human. anti MPO suitable for: IHC-P, IHC-Fr, ICC/IF, IHC-FoFr and reacts with: Mouse, Rat, Human, Pig, Monkey. anti CD68 suitable for: ICC/IF, IHC-FoFr, IHC-P, IHC-Fr and reacts with: Human. anti PCNA suitable WB, IP, IHC-P, IHC-Fr, ICC/IF, IHC-FoFr, Flow cytometry and reacts with Human, Mouse, Rat, Monkey. anti Keratin 5 suitable IHC, IHC-P, IHC-Fr, ICC, WB, Flow and reacts with Human, Mouse, Bovine. anti FOXM1 suitable WB, IP, IHC-P and reacts with Human.

anti STAT3 suitable WB, IP, Immunofluorescence, Flow cytometry, Chromatin IP, Chromatin IP-seq and reacts with Human, Mouse, Rat, Monkey. anti TNF suitable for: Flow, IHC-Fr, ICC/IF, ELISA, WB, IHC-P and reacts with: Mouse, Guinea pig, Human, Chimpanzee, Zebrafish, Cynomolgus monkey, Rhesus monkey, Apteronotus leptorhynchus. anti Ki67 suitable for: IHC - Wholemount, IHC-P, IHC-FrFl, Flow Cyt, ICC/IF, ICC and reacts with Mouse, Rat, Sheep, Rabbit, Horse, Cow, Dog, Human, Pig, Indian muntjac, Monkey, Chinese hamster, Common marmoset, Syrian hamster. anti CD45-APCCy7 suitable for: Flow cytometry and reacts with Mouse. anti CD3e-BV711 suitable for: Flow cytometry and reacts with Mouse. anti F4/80-PE suitable for: Flow cytometry and reacts with Mouse. anti CD11b-PE Dazzle 594 suitable for: Flow cytometry and reacts with Mouse. anti Ly6C-BV605 suitable for: Flow cytometry and reacts with Mouse. Ly6G- BUV395 suitable for: Flow cytometry and reacts with Mouse.

Animals and other organisms

Policy information about studies involving animals; ARRIVE guidelines recommended for reporting animal research

Laboratory animals

CD1 mice were purchased from Charles River, USA (Strain number: 022). Db/db mice were purchased from Jackson Laboratory. Both male and female mice were used in the studies. Treatments were performed at 7-9 weeks of age and all experiments were conducted using littermate controls. All animal studies were carried out according to the protocol approved by the Animal and Care Committee at the National Institute of Arthritis and Musculoskeletal and Skin Diseases and the University of Miami Miller School of Medicine. Mouse animal facilities housed mice under 12:12 light/dark cycles, with average ambient temperature of 72.9oF and 53% humidity. At the beginning of each week, complete cage change was conducted during which used (unsoiled), nesting material was transferred from the old cage to the new, clean cage. Animals were handled by the tail for all procedures. Mice were fed ad libitum and had unrestricted access to drinking water.

Wild animals

No wild animals were used.

Field-collected samples

No field collected samples were used in the study.

Ethics oversight

Animal protocols for this study were approved by the Animal and Care Committee at the National Institute of Arthritis and Musculoskeletal and Skin Diseases (protocol # A019-02-01) and by the University of Miami Institutional Animal Care and Use Committee (protocol # 18-053).

Note that full information on the approval of the study protocol must also be provided in the manuscript.

Human research participants

Policy information about studies involving human research participants

Population characteristics

Population characteristics were described in the manuscript per BRISQ guidelines; please refer to "Methods" section. Briefly, full thickness DFU (n=13, mean age±standard deviation = 56 ± 13, 13 males) and diabetic foot skin (n=S, mean age±standard deviation= 66 ±13, 7 males, 1 female) samples were obtained during initial surgical debridement from patients who fulfill: Inclusion Criteria: (1) diabetes; (2) ulcer on the plantar aspect of their foot that is larger than 0.5 cm2 and is minimum grade 1 or higher of the Wagner grading system; (3) neuropathy; (4) minimum age 21; (5) wound duration >4 weeks; 6) hemoglobin Ale 13.0%. Ulcers with clinical signs of infection were excluded. Exclusion criteria for DFU were (1) active cellulitis; (2) osteomyelitis; (3) gangrene; (4) vascular insufficiency (defined as an Ankle Brachia! Index (ABI) less than 0.7 and for those with an ABI greater than 1.3; (5) revascularization to the ipsilateral lower extremity in the last six weeks; (6) any experimental drugs taken or applied topically to the wound for 4 weeks preceding the study. For DFS samples, both non-diabetic and non-neuropathic diabetic (type 2) skin specimens were obtained from debridement procedures from patients undergoing podiatric surgery at the University of Miami Hospital.

Recruitment

Tissue specimens were obtained from discarded debridement tissue from diabetic patients with DFUs, per "Population characteristics" above. No selection biases were present; collected samples were screened for quality and suitability and those meeting thresholds were included in the study.

Ethics oversight

The protocols including written informed consent were approved by the University Institutional Review Board (IRB #20140473;#20090709). Specimens were obtained from discarded debridement procedures from patients undergoing podiatric surgery at the University of Miami Hospital and, as such, the study was found to be exempt under 45 CFR46.101.2 by the IRB at the University Of Miami Miller School Of Medicine. Specimens did not contain any of the 18 identifiers noted in the privacy rule and therefore no informed consent was obtained.

Note that full information on the approval of the study protocol must also be provided in the manuscript.

Flow Cytometry

Plots

Confirm that:

- [x] The axis labels state the marker and fluorochrome used (e.g. CD4-FITC).
- [x] The axis scales are clearly visible. Include numbers along axes only for bottom left plot of group (a 'group' is an analysis of identical markers).
- [ ] All plots are contour plots with outliers or pseudocolor plots.
- [ ] A numerical value for number of cells or percentage (with statistics) is provided.

Methodology

Sample preparation	Wound edge tissue at specific time points was collected , minced with surgical scissors and incubated for 30 minutes in dispase I solution (Roche, Basel, Switzerland)(2.4 µg/mL) at 37°C. This was followed by incubation at 37°C for 3 hours with 2 mg/ml Collagenase D (Roche) at 37°C under constant agitation. Obtained single cell suspensions were washed with IMEM (Gibco-Thermo Fisher Scientific) supplemented with 10% heat-inactivated FBS, 2 mM L -glutamine, 0.15% sodium hydrogencarbonate, 1 mM sodium pyruvate, nonessential amino acids and 50 µg/ml gentamycin. Single-cell suspensions were incubated with combinations of fluorophore-conjugated antibodies against the following surface markers: CD45-APCCy7 (30-Fll), CD3e-BV711(17A2), F4/80-PE (BMS), CDllb-PE Dazzle 594 (Ml/70), Ly6C- PerCP (HKl.4), Ly6G- BUV395 (IAS) in Hank's buffered salt solution (HBSS) for 30 min at 4°C and then washed. LIVE/DEAD Fixable Violet Dead Cell Stain Kit (Invitrogen Life Technologies) was used to exclude dead cells. All antibodies were purchased from Biolegend and BD Biosciences.
Instrument	Cell acquisition was performed on a BD LSR-Fortessa–HTS flow cytometer using FACSDiVa software (BD Biosciences).
Software	Data were analyzed using FlowJo software (TreeStar).
Cell population abundance	Cell sorting was not performed- only flow cytometry cell analysis.
Gating strategy	Flow cytometry analysis for data on Figure 7, Supplementary Figure 10, 11, 12 and 13 was performed using a forward side scattered area vs height (FSC A/H) single cell gate followed by gating on the live cells (live/dead) and then on CD45+ population within all live cells. Next, CDllb+ population was gated within all CD45+ population. These cells were then analyzed for expression of F4/80 and Ly6G. Numbers in representative plots indicate percent positive cells in each quadrant.

☒ Tick this box to confirm that a figure exemplifying the gating strategy is provided in the Supplementary Information.
